# Predicting recognition between T cell receptors and epitopes with TCRGP

**DOI:** 10.1371/journal.pcbi.1008814

**Published:** 2021-03-25

**Authors:** Emmi Jokinen, Jani Huuhtanen, Satu Mustjoki, Markus Heinonen, Harri Lähdesmäki

**Affiliations:** 1 Department of Computer Science, Aalto University, Espoo, Finland; 2 Translational Immunology Research program and Department of Clinical Chemistry and Hematology, University of Helsinki, Helsinki, Finland; 3 Hematology Research Unit Helsinki, Helsinki University Hospital Comprehensive Cancer Center, Helsinki, Finland; 4 iCAN Digital Precision Cancer Medicine Flagship, Helsinki, Finland; 5 Helsinki Institute for Information Technology, Espoo, Finland; Bar Ilan University, ISRAEL

## Abstract

Adaptive immune system uses T cell receptors (TCRs) to recognize pathogens and to consequently initiate immune responses. TCRs can be sequenced from individuals and methods analyzing the specificity of the TCRs can help us better understand individuals’ immune status in different disorders. For this task, we have developed TCRGP, a novel Gaussian process method that predicts if TCRs recognize specified epitopes. TCRGP can utilize the amino acid sequences of the complementarity determining regions (CDRs) from TCR*α* and TCR*β* chains and learn which CDRs are important in recognizing different epitopes. Our comprehensive evaluation with epitope-specific TCR sequencing data shows that TCRGP achieves on average higher prediction accuracy in terms of AUROC score than existing state-of-the-art methods in epitope-specificity predictions. We also propose a novel analysis approach for combined single-cell RNA and TCR*αβ* (scRNA+TCR*αβ*) sequencing data by quantifying epitope-specific TCRs with TCRGP and identify HBV-epitope specific T cells and their transcriptomic states in hepatocellular carcinoma patients.

## Author summary

Humans have dedicated cells called T cells that recognize potentially harmful invaders using T cell receptors (TCRs) and protect us from infections. Each person has billions of T cells and millions of different kinds of TCRs that enable us to recognize a large variety of invaders. When for example a virus enters the body, T cells with TCRs recognizing that virus multiply and fight it. After such attacks, T cells that recognized the virus, remain in the body, forming an immunological memory. T cells thus characterize a person’s infection history, but in order to reveal it, we need to determine which epitopes, molecular signatures of the invaders, different TCRs can recognize. We can characterize which TCRs recognize certain epitopes experimentally, but this is a time and sample consuming task and is not often possible with scarce patient samples such as biopsies. However, previously produced experimental data has enabled us to develop a computational method, TCRGP, that can predict which epitopes a TCR recognizes with a higher accuracy than previous methods. As a computational method, TCRGP spares resources from time and sample consuming experimental validations and offers an interesting way to analyze publicly available TCR data.

This is a *PLOS Computational Biology* Methods paper.

## Introduction

The adaptive immune system implements various complex mechanisms for surveillance against both pathogens and pathological cells arising in our body. To initiate an adequate adaptive immune response, a peptide, called epitope, must first be bound by the major histocompatibility complex (MHC) class I or II molecule expressed on the surface of a nucleated cell or a professional antigen-presenting cell, respectively. The peptide-MHC (pMHC) complex is then presented to T cells which can recognize the complex via T cell receptor (TCR) proteins, consequently leading to T cell activation and proliferation by clonal expansion [1]. During clonal expansion, a fraction of T cells gain a long-living memory phenotype and therefore a clonal population of T cells with identical TCR rearrangements remain for years against the recognized antigen [2], thus forming a potentially decodable immunological signature. Learning these signatures could have implications in broad range of clinical applications including infectious diseases, autoimmunity and tumor immunology.

T cells undergo non-homologous recombination during T cell development, which involves rearrangement of the germline TCR loci from a large collection of variable (V), diversity (D) and joining (J) gene segments as well as template-independent insertions and deletions at the V-D and D-J junctions [3, 4]. TCRs are formed by a pair of *α*- and *β*-chains (90-95% of T cells) or *γ* and *δ*-chains (5-10%) and V(D)J recombination happens in each locus independently. It is estimated that this rearrangement can result in the range of 10^18^ different TCR genes [5, 6] which provides enormous diversity for epitope-specific T cell repertoires.

The complementarity determining regions (CDRs) of a TCR determine whether the TCR recognizes and binds to an antigen or not [7]. Of these regions, CDR3 is the most variable and primarily interacts with the peptide, while CDR1 and CDR2 primarily interact with the peptide binding groove of the MHC protein presenting the peptide but they can also be directly in contact with the peptide [8, 9]. Dash et *al.* [10] noted that also a loop between CDR2 and CDR3 (IMGT positions 81-86 [11]), which they called CDR2.5, has sometimes been observed to make contact with pMHC in solved structures.

It is well known that the CDR3*β* of a TCR is important in recognizing peptides presented to the T cell, but it still remains unclear which specific physicochemical or structural features of the CDR3*β* or of other parts of the TCR determine the antigen recognition specificity of the T cell. High-throughput sequencing of V- and J-segment enriched DNA has enabled large-scale characterization of TCR sequences, initially only for the CDR3*β* with bulk methods [6, 12] but recently for the whole paired TCR*αβ* at single-cell resolution using plate or droplet-based methods [13, 14]. Nevertheless, profiling of epitope-specific TCRs remains exhaustive as they require sample-consuming experiments with distinct pMHC-multimers for each epitope of interest. Therefore, there is a great need for models that examine which epitopes a TCR can recognize or to which TCRs an epitope can bind to [15]. Curated databases of experimentally verified TCR-peptide interactions have recently been launched, such as VDJdb, IEDB, and McPAS [16–18]. Such data sources enable more comprehensive, data-driven analysis of TCR-peptide interactions, and allow the use of statistical machine learning techniques for the aforementioned tasks. Yet only a few computational methods for predicting recognition between TCRs and epitopes [10, 19–22] and for clustering similar TCRs [9, 23, 24] have been published. In addition to supervised and unsupervised methods for predicting TCR-epitope interactions, computational methods and web services such as [25] have also been proposed to predict the structure of TCRs based on their amino acid sequences.

We propose a method called TCRGP which builds on non-parametric modelling using Gaussian process (GP) classification. The probabilistic formulation of GPs allows robust model inference already from small data sets, which is a great benefit as currently there exists very limited amounts of reported TCR-epitope interactions in curated databases. As the space of all TCRs that can recognize a certain epitope is potentially very large, it is important to avoid overfitting to the limited sample of TCRs that is available. Indeed, TCRGP clearly outperforms the current state-of-the art methods for predicting the epitope specificity of TCRs. At the same time TCRGP can scale to exploit extremely large data sets of epitope-specific TCRs, which we expect to become more common in the future. We also analyze the effects of utilizing different sections of the TCR amino acid sequence and learn which of them are most important and examine how the number of TCRs used for training affects the predictions. Finally, we demonstrate the potential of TCRGP by analyzing single-cell RNA+TCR*αβ*-sequencing data from hepatocellular carcinoma patients.

## Results

### Gaussian process classifier

Gaussian processes (GP) are a flexible class of models that have become popular in machine learning and statistics with various applications in molecular biology, bioinformatics and other fields [26–30]. We have developed TCRGP, a GP based probabilistic classifier which can be trained to predict TCRs’ specificity to any epitope given sufficient training data for the specified epitope. GPs implement a Bayesian nonparametric kernel method for learning from data. They differ from standard parametric models in that instead of learning the parameters of a predefined function, they define priors for the entire class of nonlinear functions and learn a suitable function for the prediction task. Properties of GPs are defined by the kernel function, which is a function of objects that we want to classify. TCRGP uses Gaussian process classification with variational inference [31, 32]

**Fig 1:**
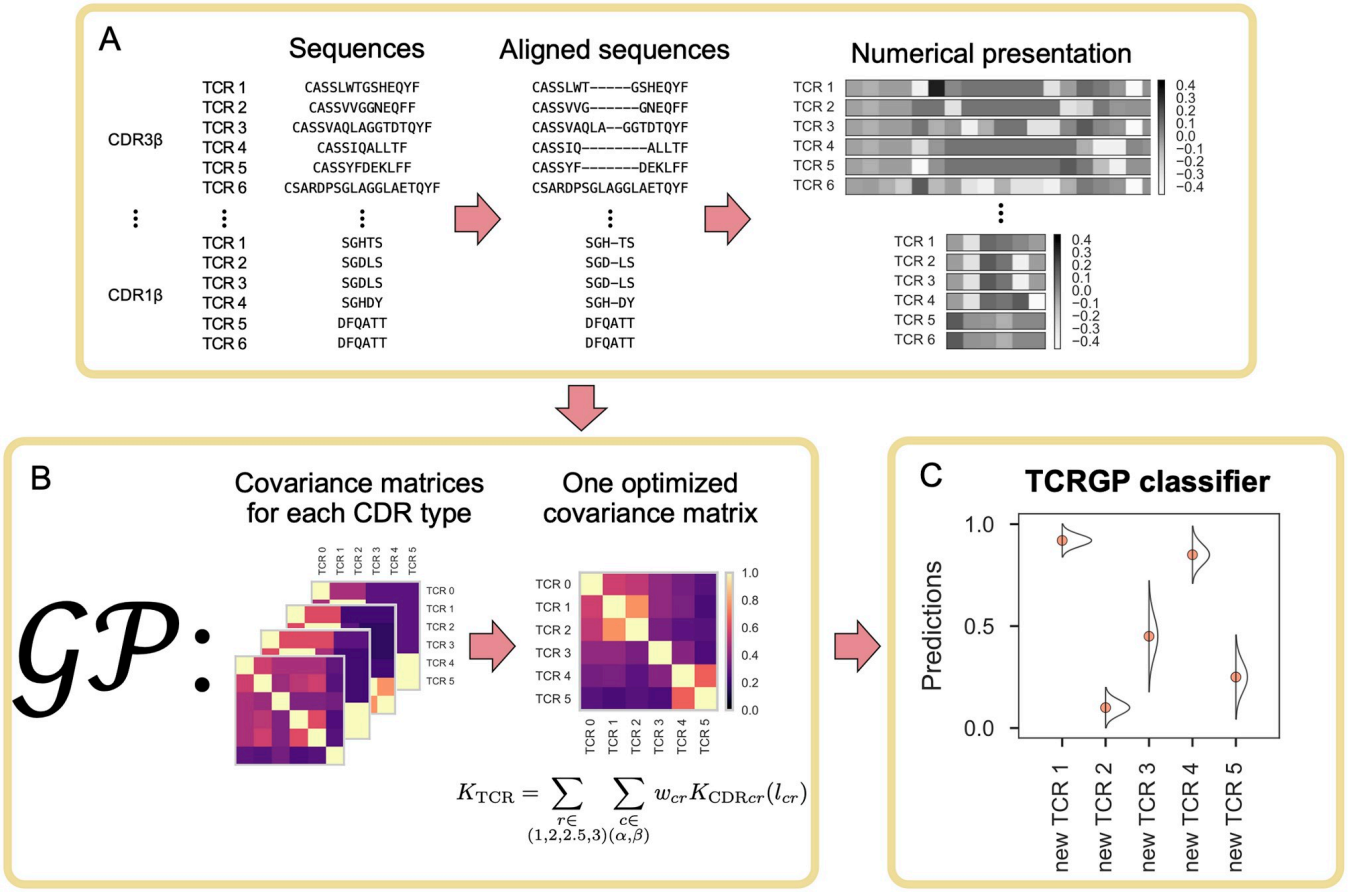
TCRGP pipeline for training a classifier to predict if new unseen TCRs recognize a certain epitope. **(A)** Sequence preparation. For the training, TCRs specific to the epitope of interest and control sequences not recognizing the epitope are required. TCRGP can utilize CDR3, CDR1, CDR2, and CDR2.5 sequences from both TCR *α*- and *β*-chains. A separate alignment is created for each CDR type and the aligned sequences are given numerical presentations. We use principal components of a modified BLOSUM62 substitution matrix to encode each amino acid. We utilize all 21 components, but in this illustration we only show the first components. **(B)** Using the numerical presentation, we create separate covariance matrices for each CDR type. During the training of the Gaussian process classifier, an optimal combination of the base kernels and their parameters are learned. **(C)** When the classifier has been trained, we can make probabilistic predictions for new TCRs.

To train a GP classifier for predicting if TCRs recognize a certain epitope, a set of training data consisting of TCRs that are known to recognize and to not recognize that epitope are required. TCRGP can utilize CDR3 amino acid sequences from both TCR *α*- and *β*-chains as well as CDR1, CDR2, and CDR2.5 sequences which can be determined from V*α*- and V*β*-genes. The different CDRs are then aligned within each CDR type using the IMGT definitions and a numerical presentation of them is formed using principal components of a modified BLOSUM62 substitution matrix (see Fig 1A).

Using the numerical presentations, a separate covariance matrix is created for each CDR type. The covariance matrices measure similarity between all pairs of TCRs (separately for each CDR type). The GP then learns optimal parameters for the kernel functions that define the covariance matrices for each CDR type and learns simultaneously an optimal combination of these kernel functions. As the model can determine itself the weights for each CDR type, it can learn which CDRs are important for the classification task. Due to the probabilistic nature of GPs, we can use marginal likelihood maximisation for the optimisation and do not require additional data for validating the learned parameters. This is a great benefit, when there exists limited amounts of epitope-specific TCRs, which is currently the case with many epitopes. Moreover, by using sparse variational inference, the GPs can also scale to extremely large data sets when more data is available (see Fig 1B).

Once the TCRGP classifier has been trained, it can be used to provide predictions to new, previously unseen, TCRs. The certainty of the predictions is encoded in the predicted values: If the prediction for a TCR is close to one, it is very likely to recognize the epitope in question, if it is close to zero, the TCR very likely does not recognize the epitope. However, if the prediction is around 0.5, the model cannot give a prediction with much certainty to that TCR (see Fig 1C). A more detailed description of TCRGP can be found from the Materials and Methods section.

We use two data sets to demonstrate TCRGP’s accuracy in predicting TCR epitope specificity: a recently published data set of tetramer sorted TCR sequences for 10 epitopes, introduced by Dash et *al.* [10] (Dash data), and a new dataset of medium and high quality epitope-specific TCR sequences extracted from VDJdb database [16] (VDJdb data). The Dash data provides a large set of epitope-specific paired TCR*αβ*-data and the VDJdb data provides a comprehensive selection of available epitope-specific TCR*β*-data currently available. We also considered using using TCRs from IEDB [17] and McPAS [18], but they had significant overlap with VDJdb and their collections of TCR*β*s were not as extensive. Both of the selected data sets are combined with a set of background TCRs, also presented by Dash et *al.* [10] that are not expected to recognize the epitopes in the two data sets. Our work is accompanied by an efficient software implementation that contains trained models for predicting TCRs’ specificity to epitopes involved in data sets used in this study as well as tools for building new epitope specificity models from new datasets. The implementation and used data sets are available at github.com/emmijokinen/TCRGP.

### Significance of utilizing different CDRs

**Fig 2:**
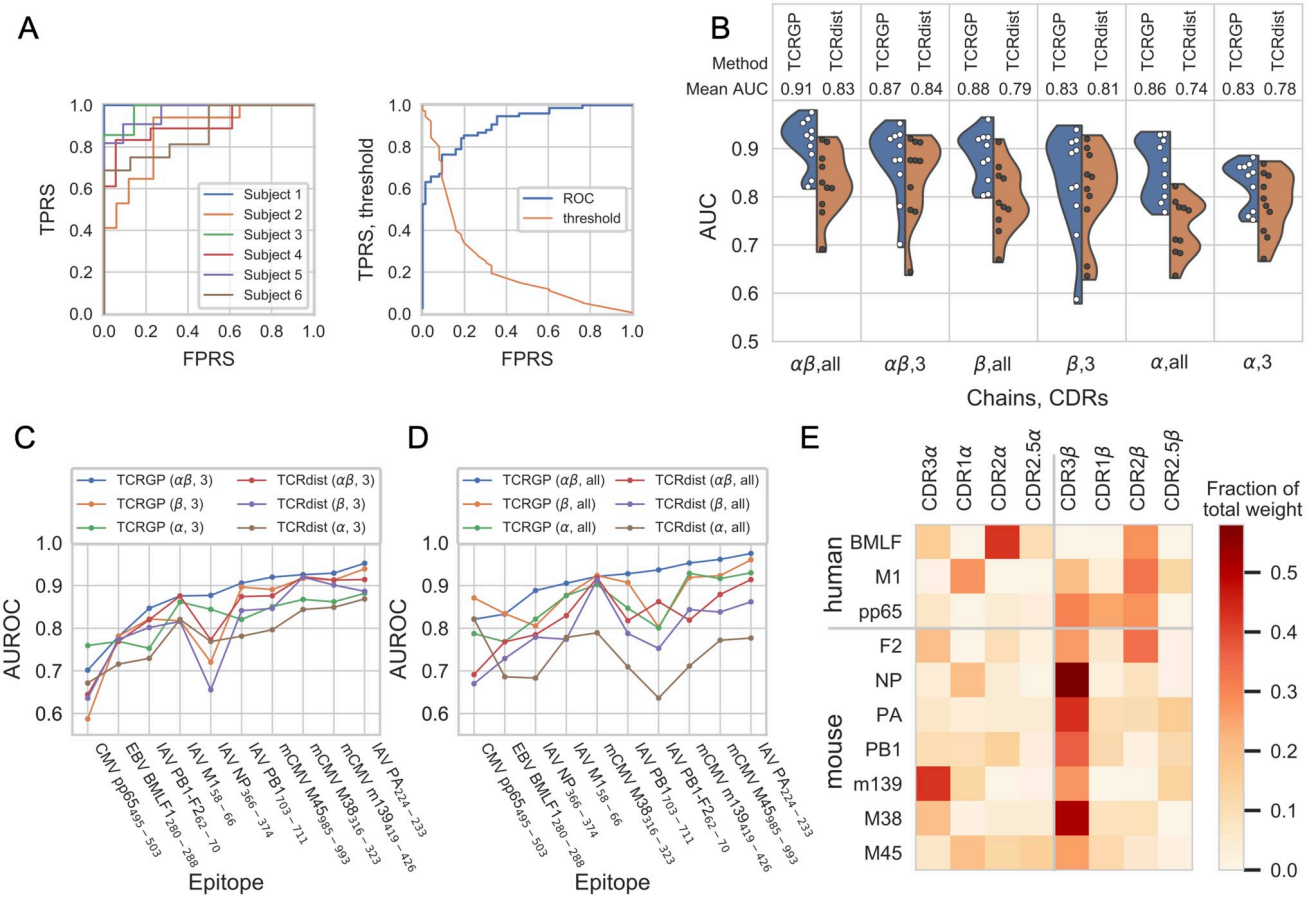
Epitope-specificity prediction with the Dash data. **(A)** The left panel shows the cross-validated ROC curves for each subject in the Dash data for BMLF1_280-288_, when TCRGP has been trained using all CDRs from TCR*α* and TCR*β*. The mean AUROC score is 0.905. The right panel shows the cross-validated ROC curve for all subjects and also the corresponding threshold values. From this figure we can determine which threshold values correspond to different true positive rates (TPRS) and false positive rates (FPRS). **(B)** Violin plots present the distributions of AUROC scores for the different epitopes obtained with models utilizing varying CDRs. The blue parts of the violin plots illustrate the AUROC scores of predictions made by TCRGP for all the epitopes. The orange sides illustrate the AUROC scores obtained with TCRdist. Each point within a violin plot presents the mean AUROC score obtained for one epitope. The used chains (*α* and/or *β*) and CDRs (three or all) are indicated below each panel. **(C)** Comparison of AUROC scores obtained with TCRGP and TCRdist using only CDR3 from TCR*αβ*, TCR*β*, or TCR*α* for each epitope separately. The epitopes have been arranged in increasing order of AUROC scores obtained by TCRGP using CDR3 from *α*- and *β*-chains (blue line). **(D)** Comparison of AUROC scores obtained with TCRGP and TCRdist using all CDRs from TCR*αβ*, TCR*β*, or TCR*α* for each epitope separately. The epitopes have been arranged in increasing order of AUROC scores obtained by TCRGP using all CDRs from *α*- and *β*-chains (blue line). **(E)** Fractions of total weight given to kernels corresponding to different CDRs, when TCRGP has been trained to predict which TCRs are specific to the epitopes in the Dash data using all CDRs from both TCR chains.

To evaluate the benefit of using different CDRs, we used the Dash data which includes 4635 pMHC-tetramer sorted single-cell sequenced TCR*αβ* clonotypes from 10 epitope-specific repertoires. We trained our TCRGP model using either only CDR3 or also with CDR1, CDR2, and CDR2.5 from TCR*α*, TCR*β*, or both. We applied leave-one-subject-out cross-validation as described in Materials and Methods Section. Fig 2A presents the cross-validation results for a single BMLF1_280-288_-epitope from EBV and demonstrates how the classification results vary between different subjects likely due to the variety of the TCRs.

AUROC scores of the predictions for different combinations of CDRs and *α*/*β* chains are summarized in Fig 2B. For comparison, we also trained TCRdist [10] in the same manner. Fig 2B shows that both methods, TCRGP and TCRdist, perform on average better when using TCR*β* than when using TCR*α*, although using both *α*- and *β*-chains generally provides the best results. There are few exceptions, as shown in Fig 2B–2D and S1 Fig and Table 1. For example, with epitope pp65 both methods perform better when using CDR3*α* instead of CDR3*β*. Overall TCRGP is better than TCRdist in utilizing information from CDRs other than CDR3. TCRGP achieves higher AUROC scores on average when trained using all CDRs instead of only CDR3, whereas with TCRdist the AUROC scores seem to be similar or better when only CDR3 is utilized. Notably TCRGP outperforms TCRdist in prediction accuracy for 57 of the 60 comparisons (Table 1 and S1 Fig). To quantify the significance of accuracy differences between different methods, we applied Wilcoxon signed-rank test to paired AUROC scores across epitopes. We used the test to study three questions: do AUROC scores differ between TCRGP and previous methods, is it better to use all CDRs or only CDR3s, and which chains should be used to produce best results. The Benjamini-Hochberg corrected P-values for these tests can be found from S2 File. These results support the above observations: TCRGP outperforms TCRdist, with TCRGP it is beneficial to use all CDRs, and the use of *β* chain generally gives better AUROC scores than *α* chain, although it is best to use both chains. It is noteworthy, that although Dash et *al.* [10] use leave-one-subject-out control, the results shown here differ somewhat from their results. The differences are likely caused by differences in the background TCRs as we have selected them randomly from the larger set of background TCRs provided by Dash et *al.*

**Table 1:** Mean AUROC scores for the Dash data using leave-one-subject-out cross-validation. TCRGP models and TCRdist models were trained using either only CDR3s or all CDRs from TCR*αβ*, TCR*β*, or TCR*α*.

	Method	TCRGP						TCRdist					
	chains	*αβ*		*β*		*α*		*αβ*		*β*		*α*	
	cdrs	3	all	3	all	3	all	3	all	3	all	3	all
	mean	0.871	0.913	0.830	0.883	0.823	0.856	0.834	0.828	0.807	0.797	0.781	0.732
EBV	BMLF1_280-288_	0.876	0.906	0.818	0.877	0.862	0.877	0.875	0.830	0.816	0.774	0.821	0.778
CMV	pp65_495-503_	0.702	0.821	0.587	0.871	0.759	0.787	0.645	0.691	0.636	0.670	0.672	0.822
IAV	M1_58-66_	0.781	0.833	0.782	0.834	0.769	0.768	0.769	0.768	0.774	0.729	0.716	0.686
IAV	PB1-F2_62-70_	0.847	0.937	0.822	0.803	0.753	0.801	0.820	0.862	0.802	0.753	0.729	0.636
IAV	NP_366-374_	0.906	0.928	0.897	0.907	0.821	0.847	0.874	0.818	0.841	0.788	0.781	0.709
IAV	PA_224-233_	0.952	0.975	0.939	0.961	0.882	0.930	0.914	0.914	0.887	0.862	0.869	0.777
IAV	PB1_703-711_	0.929	0.953	0.913	0.919	0.862	0.929	0.913	0.819	0.901	0.844	0.849	0.711
mCMV	m139_419-426_	0.877	0.888	0.721	0.806	0.844	0.821	0.773	0.785	0.656	0.779	0.769	0.683
mCMV	M38_316-323_	0.926	0.922	0.917	0.924	0.868	0.902	0.921	0.918	0.920	0.914	0.844	0.789
mCMV	M45_985-993_	0.920	0.962	0.891	0.923	0.851	0.916	0.876	0.879	0.846	0.838	0.796	0.772

Fig 2B–2D, Table 1, and S1 Fig also show that the AUROC scores can have notable differences between different epitopes even when the same combinations of CDRs and *α*/*β* chains have been utilized. Some of these differences may be explained by the differences in the number of available training samples, for example for CMV-epitope pp65_495-503_ there were only 76 TCRs from 6 subjects in the Dash data, which may have contributed to a lower prediction accuracy. To address this, we evaluated the models also using leave-one-out cross-validation with only unique, private TCRs to see how the models perform when predictions are done only on new TCRs. With both TCRGP and TCRdist, the average AUROC scores improve slightly (S3 Fig), demonstrating that the models can predict if completely new TCR sequences are specific to these epitopes and that the larger number of TCRs used for training (due to the larger folds in leave-one-out cross validation) improve the model performances. A summary of all prediction results is shown in S1 File.

To better understand the significance of the different CDRs for TCR-pMHC recognition, we also examined more closely how TCRGP weighted the kernels created for the different CDRs, when all CDRs from both chains were utilized. Fig 2C illustrates which CDRs were found important for the different epitopes. As one might expect, with most of the epitopes most weight was given to the CDR3*β*, but with all epitopes some weight was also given to other CDRs, suggesting that the classifier benefits from utilizing several CDRs. This is in agreement with an alignment of 52 TCR sequences from TCR-pMHC PDB structure complexes, which demonstrates that all CDRs can be within 5Å of a peptide [9]. For example with CMV-epitope pp65_495-503_, experimental characterization of the structure (PDBid 3GSN) showed CDR3*β*, CDR1*β*, CDR3*α* and CDR1*α* to be within 5Å of the peptide, and also CDR2*β* within 5.8Å of the peptide. Another TCR-pMHC-complex structure (PDBid 5D2L) for the same pp65_495-503_-epitope suggests that CDR2*β* was also within 5 Å of the peptide. Indeed, the optimized weights for the pp65_495-503_-epitope (Fig 2C) show some correspondence to the observed contacts. Although with many epitopes CDR3*β* was considered as the most important CDR, this was not the case with all epitopes. For example with mCMV-epitope m139_419-426_ CDR3*α* is more important for the prediction, while with EBV-epitope BMLF1_280-288_ most of the weight was given to CDR2*α* and CDR2*β*. Still, also with BMLF1_280-288_ epitope the CDR3*β* alone provided discriminative information, as demonstrated by the high AUROC score (0.818) when TCRGP was trained only with CDR3*β*-sequences (see Table 1 or S1 Fig).

### Comparisons to other methods

Currently only a few methods for predicting the epitope-specificity of TCRs have been published, including TCRdist [10] and a random forest (RF) classifier by De Neuter et *al.* [19]. TCRdist uses a BLOSUM62 based distance measure between amino acids and defines the distance between two (aligned) TCRs as a weighted sum over the amino acid distances. Using this distance measure, they determine if a TCR is closer to an epitope-specific cluster of TCRs or to a cluster of background TCRs. De Neuter et *al.* use feature vectors formed by biophysical and other features extracted from TCR amino acid sequences to train a RF classifier. Gielis et *al.* [33] have later published a web server TCRex that is based on the RF classifier. However, we have utilized the implementation from the original article to be able to train models with the same training sets as with the other models in the comparisons. More recently also methods relying on neural networks have been proposed, such as DeepTCR, NetTCR, and Ergo [20–22]. DeepTCR relies on convolutional networks and uses one-hot encoding to present CDR3 and V/D/J-genes from *α*- and *β*-chains, or a subset of these, to learn embeddings for the sequences. NetTCR, which is intended for HLA-A*02:01 restricted epitopes, also uses convolutional neural networks, but utilizes BLOSUM50 encoding for presenting the CDR3*β* and the epitope sequence. Ergo uses LSTM (long-short term memory) network to encode the peptide sequence and either LSTM or an autoencoder to encode the CDR3 and utilize feed forward networks for predictions. Here we provide comparisons between TCRGP, TCRdist, RF, and DeepTCR.

**Fig 3:**
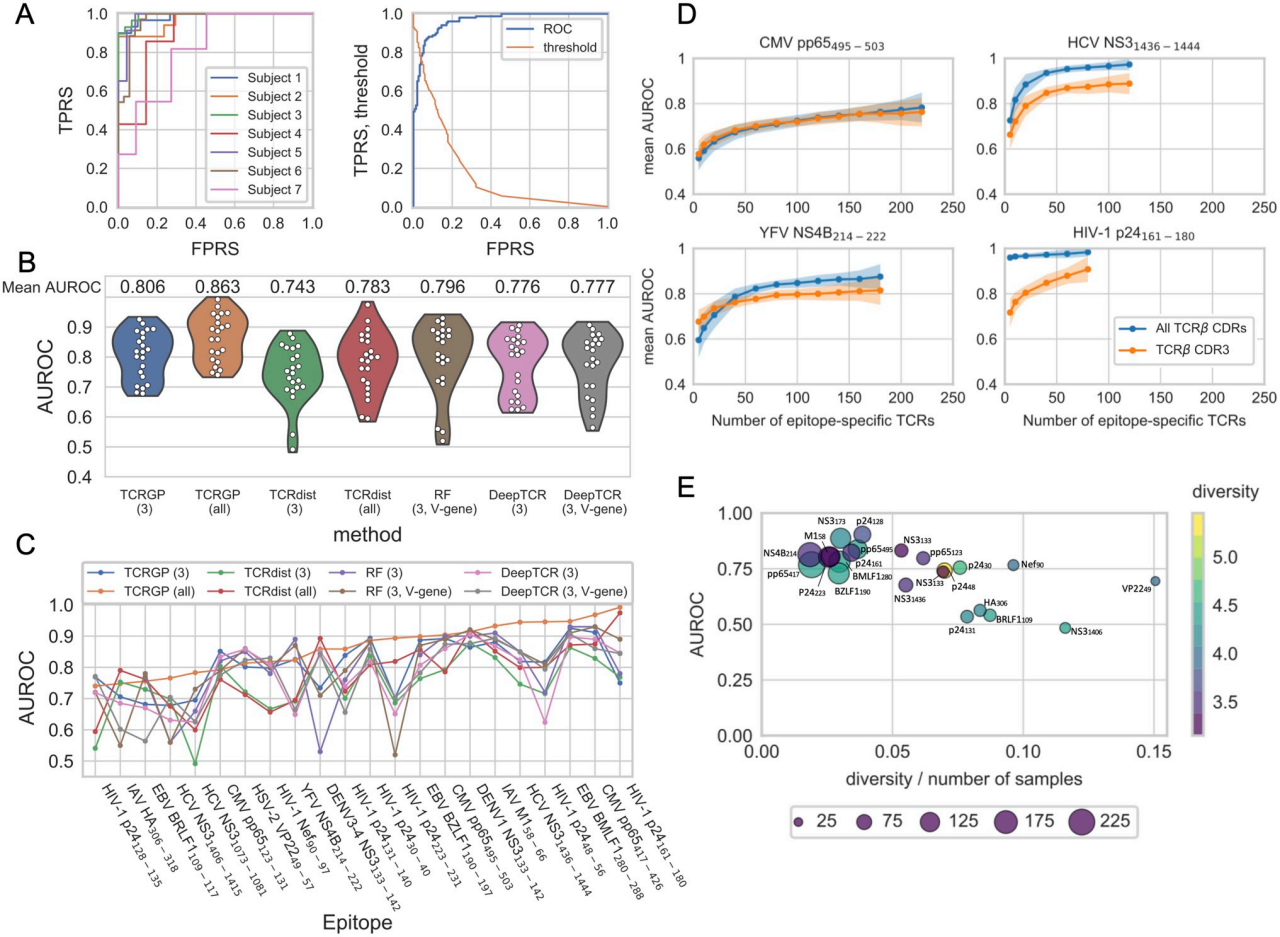
Epitope specificity prediction with the VDJdb data. **(A)** The left panel shows the cross-validated ROC curves for each subject in the VDJdb data for HCV NS3_1436-1444_-epitope, when TCRGP has been trained using TCR*α* and TCR*β* with all CDRs. The mean AUROC score is 0.944. The right panel shows the cross-validated ROC curve for all subjects and also the threshold values for classification are shown. From this figure we can determine which threshold values correspond to different true positive rates (TPRS) and false positive rates (FPRS). **(B)** One violin plot presents the distribution estimate of mean AUROC scores obtained with one method for all epitopes in our VDJdb data. Below each violin plot there is the name of the method used and in the brackets which CDR*β*s have been used (3 for CDR3, all for CDR1, CDR2, CDR2.5, and CDR3). Each point within a violin plot presents the mean AUROC score obtained for one epitope. RF refers to the Random Forest TCR-classifier of De Neuter et *al.* [19]. RF using only CDR3*β* has not been included in this figure as it could not provide predictions for all of the 22 epitopes. **(C)** Comparison of AUROC scores obtained with the different methods for each epitope separately. The epitopes have been arranged in increasing order of AUROC scores obtained by TCRGP using all CDR*β*s (orange line) **(D)** For each epitope from the VDJdb dataset, TCRGP models were trained using different numbers of unique epitope-specific TCR*β*s, always complemented with the same number of control TCR*β*s. For each point of the learning curve the model was trained with 100 random samples of the TCR*β*s, using either CDR1, CDR2, CDR2.5, and CDR3 (blue curves), or only CDR3 (orange curves). The darker lines show the mean of the predictions and the shaded areas ± the standard deviation for the 100 folds. The points indicate the tested sample sizes. Here learning curves for four peptides are shown. **(E)** Leave-one-out cross-validated AUROC scores correlate with the diversity and number of samples (Pearson correlation -0.66). The sizes of the circles indicate the number of unique TCRs used for training.

To compare TCRGP to these other methods, we experimented with the VDJdb data and we again used leave-one-subject-out cross-validation as described in Materials and Methods Section. We trained TCRGP and TCRdist using only CDR3*β* and then also with the other CDR*β*s. Fig 3A shows the ROC curves when TCRGP was trained with all CDR*β*s to predict which TCRs are specific to the HCV-epitope NS3_1436-1444_.

RF and DeepTCR also utilize the CDR3*β* sequence, but use V*β*-gene instead of the other CDR*β*s. RF could also utilize the J-gene and DeepTCR J- and D-genes, but unfortunately, the background TCR data set from Dash et *al.* [10] did not contain information of these genes. However, according to De Neuter et *al.*, not much weight was given to the J-gene at least in their experiments. Moreover, when RF and DeepTCR utilize CDR3*β* and V*β*-gene and TCRGP and TCRdist use all CDRs, all four methods get the same sequence information, although in a slightly different form, as the other CDR*β*s are derived from the V*β*-gene.

Fig 3B shows the distribution estimates of mean AUROC scores for each model trained for the 22 different epitopes. With the VDJdb data, we can see that TCRGP and TCRdist both perform better, when all CDR*β*s have been utilized. Remarkably, TCRGP achieves higher mean AUROC scores than the other methods when using all CDRs, but also when only the CDR3*β* is utilized. AUROC scores for the different epitopes are presented in Table 2 and S4 Fig and Fig 3C illustrate how the AUROC scores vary between different methods separately for each epitope. The epitopes have been arranged in increasing order of AUROC scores obtained by TCRGP using all CDR*β*s. We can see that with most epitopes there are large differences in the accuracies of the different methods.

**Table 2:** Mean AUROC scores for the VDJdb data using leave-one-subject-out cross-validation. TCRGP models and TCRdist models were trained using TCR*β* with either only CDR3*β* or all CDR*β*s. RF models and DeepTCR models were trained using only CDR3*β* or CDR3*β* with V*β*-gene, from which the other CDR*β*s can be derived from.

	Method	TCRGP		TCRdist		RF		DeepTCR	
	CDRs	3	all	3	all	3	3, V-gene	3	3, V-gene
	mean	0.806	0.863	0.743	0.783	NaN	0.796	0.776	0.777
CMV	pp65_123-131_	0.852	0.792	0.804	0.761	0.820	0.790	0.834	0.775
CMV	pp65_417-426_	0.912	0.968	0.829	0.874	0.930	0.930	0.890	0.860
CMV	pp65_495-503_	0.892	0.904	0.793	0.786	0.900	0.890	0.860	0.874
EBV	BMLF1_280-288_	0.926	0.947	0.863	0.872	0.930	0.910	0.897	0.907
EBV	BZLF1_190-197_	0.887	0.899	0.764	0.857	0.840	0.870	0.807	0.782
EBV	BRLF1_109-117_	0.682	0.756	0.729	0.762	0.770	0.780	0.670	0.564
IAV	M1_58-66_	0.881	0.933	0.832	0.852	0.910	0.890	0.868	0.893
IAV	HA_306-318_	0.706	0.748	0.753	0.790	NaN	0.550	0.685	0.602
HCV	NS3_1073-1081_	0.695	0.783	0.492	0.600	0.660	0.730	0.625	0.626
HCV	NS3_1406-1415_	0.678	0.766	0.698	0.676	0.560	0.560	0.631	0.704
HCV	NS3_1436-1445_	0.819	0.945	0.746	0.799	0.850	0.850	0.824	0.849
HSV-2	VP22_49-57_	0.801	0.814	0.721	0.713	0.850	0.860	0.858	0.823
YFV	NS4B_214-222_	0.825	0.823	0.692	0.695	0.890	0.870	0.649	0.664
DENV1	NS3_133-142_	0.864	0.914	0.878	0.920	0.900	0.920	0.906	0.876
DENV3-4	NS3_133-142_	0.734	0.859	0.842	0.893	0.530	0.710	0.850	0.843
HIV-1	p24_30-40_	0.894	0.886	0.836	0.808	0.880	0.880	0.818	0.858
HIV-1	p24_48-56_	0.817	0.946	0.716	0.800	0.720	0.810	0.624	0.794
HIV-1	p24_128-135_	0.770	0.740	0.541	0.594	0.720	0.720	0.719	0.771
HIV-1	p24_131-140_	0.838	0.859	0.701	0.724	0.760	0.790	0.740	0.656
HIV-1	p24_161-180_	0.750	0.992	0.769	0.975	0.780	0.890	0.846	0.844
HIV-1	p24_223-231_	0.702	0.894	0.686	0.819	NaN	0.520	0.651	0.703
HIV-1	Nef_90-97_	0.798	0.818	0.667	0.657	0.780	0.800	0.809	0.830

In the VDJdb data, there were also TCRs that appeared in samples collected from multiple subjects (see Table 3). We therefore trained the models also using leave-one-out cross-validation with only unique TCRs. In this case we considered a TCR to be unique if it had a unique combination of CDR3*β* amino acid sequence and V*β*-gene, as we only utilized the TCR*β*. As with the Dash data above, our results (S5 and S6 Figs) show that the models can predict if completely new sequences are specific to these epitopes, thus demonstrating their use for epitope specificity prediction for previously unseen TCRs. A summary of all prediction results is shown in S1 File.

**Table 3:** Datasets. Dash data: The data set constructed by Dash et *al.* [10] contains epitope-spcefic TCRs for Epstein-Barr virus (EBV), human Cytomegalovirus (CMV), Influenza A virus (IAV) and mouse Cytomegalovirus (mCMV). VDJdb data: Data set gathered from VDJdb contains epitope-specific TCRs for Cytomegalovirus (CMV), Epstein-Barr virus (EBV), Influenza A virus (IAV), Hepatitis C virus (HCV), Herpes Simplex virus type 2 (HSV-2), Yellow Fever virus (YFV), Dengue virus type 1 (DENV1), Dengue virus type 3 (DENV3-4), and Human immunodeficiency virus type 1 (HIV-1). For the MHC chains we show here only the allele group. For some epitopes there are TCRs for which there exists more detailed information and some variation, which are shown in S3 File.

Dash data								
Species	Epitope species	Epitope gene	Epitope	MHC chain 1	MHC chain 2	Subjects	Samples	Unique TCR*αβ*s
Human	EBV	BMLF1_280-288_	GLCTLVAML	HLA-A*02:01	-	6	76	69
	CMV	pp65_495-503_	NLVPMVATV	HLA-A*02:01	-	10	61	60
	IAV	M1_58-66_	GILGFVFTL	HLA-A*02:01	-	15	275	237
Mouse	IAV	PB1-F2_62-70_	LSLRNPILV	D^b^	-	9	117	117
	IAV	NP_366-374_	ASNENMETM	D^b^	-	24	305	263
	IAV	PA_224-233_	SSLENFRAYV	D^b^	-	15	324	293
	IAV	PB1_703-711_	SSYRRPVGI	K^b^	-	34	642	584
	mCMV	m139_419-426_	TVYGFCLL	K^b^	-	8	87	87
	mCMV	M38_316-323_	SSPPMFRV	K^b^	-	14	158	143
	mCMV	M45_985-993_	HGIRNASFI	D^b^	-	13	291	271
VDJdb data								
Human	CMV	pp65_123-131_	IPSINVHHY	HLA-B*35	B2M	17	65	58
	CMV	pp65_417-426_	TPRVTGGGAM	HLA-B*07	B2M	29	184	122
	CMV	pp65_495-503_	NLVPMVATV	HLA-A*02	B2M	103	413	242
	EBV	BMLF1_280-288_	GLCTLVAML	HLA-A*02	B2M	54	299	152
	EBV	BZLF1_190-197_	RAKFKQLL	HLA-B*08	B2M	17	225	149
	EBV	BRLF1_109-117_	YVLDHLIVV	HLA-A*02	B2M	6	66	51
	IAV	M1_58-66_	GILGFVFTL	HLA-A*02	B2M	50	239	138
	IAV	HA_306-318_	PKYVKQNTLKLAT	HLA-DRA*01	HLA-DRB1*01,04	11	56	50
	HCV	NS3_1073-1081_	CINGVCWTV	HLA-A*02	B2M	7	76	39
	HCV	NS3_1406-1415_	KLVALGINAV	HLA-A*02	B2M	4	65	65
	HCV	NS3_1436-1445_	ATDALMTGY	HLA-A*01	B2M	7	152	139
	HSV-2	VP22_49-57_	RPRGEVRFL	HLA-B*07	B2M	5	68	29
	YFV	NS4B_214-222_	LLWNGPMAV	HLA-A*02	B2M	5	223	198
	DENV1	NS3_133-142_	GTSGSPIVNR	HLA-A*11	B2M	11	65	59
	DENV3-4	NS3_133-142_	GTSGSPIINR	HLA-A*11	B2M	8	51	46
	HIV-1	p24_30-40_	KAFSPEVIPMF	HLA-B*57	B2M	44	134	104
	HIV-1	p24_48-56_	TPQDLNTML	HLA-B*42,81	B2M	21	52	40
	HIV-1	p24_128-135_	EIYKRWII	HLA-B*08	B2M	12	81	60
	HIV-1	p24_131-140_	KRWIILGLNK	HLA-B*27	B2M	27	212	141
	HIV-1	p24_161-180_	FRDYVDRFYKTLRAEQASQE	HLA-DRA*01	HLA-DRB1*01,07,11,15, HLA-DRB5*01	17	141	95
	HIV-1	p24_223-231_	GPGHKARVL	HLA-B*07	B2M	1	62	53
	HIV-1	Nef_90-97_	FLKEKGGL	HLA-B*08	B2M	21	104	78

To affirm the above observations, we again applied Wilcoxon signed-rank test. We used the test to study two questions: do AUROC scores differ between TCRGP and previous methods, and is it better to use all CDR*β*s rather than only CDR3*β*s. The Benjamini-Hochberg corrected P-values for these tests (see S2 File) support the visual observations we made from Fig 3B.

### Significance of the number of training samples

To assess how the number of epitope-specific TCRs affects the performance of TCRGP classifier, we trained our model using different numbers of epitope-specific TCRs from the VDJdb data. We selected all unique TCRs for each epitope and took 100 random samples from them for each training set size and used the remaining TCRs for testing. The epitope-specific TCRs in the training and test sets were accompanied with equal numbers of randomly chosen control TCRs. Learning curves for four epitopes are shown in Fig 3D and learning curves for all 22 epitopes in S7 Fig. In general, the predictive performance of the TCRGP classifiers improve when more training samples are available. Indeed, we observed a negative correlation between TCRs’ diversity and sample size ratio and prediction accuracy (Fig 3E). We also examined if the diversities of epitope-specific TCRs within and between subjects differ. We found that although the diversities of TCRs specific to different epitopes vary, on average diversities within and between subjects are similar (panel B in S8 Fig). However, there is still variation in the diversities between pairs of subjects (panel C in S8 Fig).

These learning curves also further demonstrate the benefit of using multiple CDR sequences: With most of the epitopes using all CDRs produces better or comparable AUROC scores with all sample sizes, although there are a few epitopes with which the AUROC scores are higher when utilizing only the CDR3*β* if the sample sizes are very small (≤40). These results also suggest that with many epitopes it may be more beneficial to sequence a moderate amount of TCRs in such precision that in addition to the CDR3 also the V-gene and allele (and thus the CDR1, CDR2, and CDR2.5) can be determined, than to sequence large amounts of only CDR3s. These findings are in line with the weights learned by TCRGP for each CDR3 for the individual epitopes, as we can see in the case of CMV-epitope pp65_495-503_, EBV-epitope BMLF1_280-288_ and IAV-epitope M1_58-66_ (see Fig 2). With pp65_495-503_ most weight was given to CDR3*β* and thus information from other CDRs was not considered as beneficial; with BMLF1_280-288_ almost no weight was given to CDR3*β* and in the learning curves there is a clear improvement when all CDR*β*s are used; with M1_58-66_ some weight was given to CDR3*β*, but most weight fell to CDR2*β* and correspondingly there is a small improvement in the learning curves, when all CDR*β*s are utilized. Overall, the learning curves show that TCRGP can learn an accurate predictor even from a small data set, thus making it applicable to the currently existing TCR-peptide interaction data sets. On the other hand, our results also show that TCRGP’s prediction accuracy increases along with increasing number of training examples, enabling analysis of larger TCR-peptide interaction data sets in the future.

### Discriminating between epitope-specific TCRs

**Fig 4:**
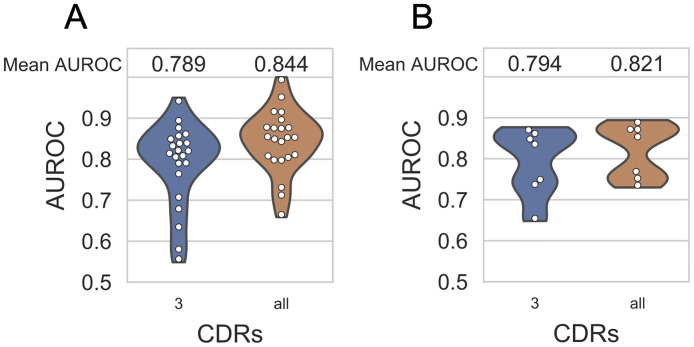
Mean AUROC scores obtained from TCRGP models with different kinds of data. Blue violin plots show results of models trained with only CDR3*β* and orange violin plots of models trained with all CDR*β*s. **(A)** Mean AUROC scores from TCRGP models for the 22 epitopes in VDJdb data, when TCRs specific to one epitope are considered as positive and TCRs specific to the other 21 epitopes are considered as control data. 200-fold stratified cross validation was used for the evaluation. **(B)** Same as (A), but only the 885 unique TCRs specific to the seven HLA-A*02 restricted epitopes are used.

To further validate the performance of TCRGP, we also tested it in a setting were we use only TCRs from the VDJdb data so that TCRs specific to one epitope are considered as positive and TCRs specific to the other epitopes are considered as negative. We only utilized unique TCRs for each epitope and used stratified 200-fold cross validation. We observed some cross-reactivity between TCRs specific to some of the epitopes, for example DENV1 epitope NS3_133-142_ and DENV3-4 epitope NS3_133-142_ have many TCRs that are specific to both epitopes. When each of the models were trained, we therefore excluded TCRs from the control data that were known to recognize the epitope in question. Fig 4A shows that TCRGP performs well also with this kind of data, as the mean AUROC scores are very similar to those achieved when using leave-one-out cross-validation and the control data from Dash et *al.* [10] (see S5 and S9 Figs).

The VDJdb data we have used contains epitopes restricted by MHCs with different HLA types. Structural data from TCR-pMHC complexes indicate that CDR1 and CDR2 are likely to interact more with the MHC than with the epitope. To see if using other CDR*β*s than CDR3*β* is beneficial also when all epitope-specific and control TCRs can recognize epitopes restricted by MHCs of the same HLA-type, we performed the above test with only TCRs specific to HLA-A*02 restricted epitopes (see Table 3 and S3 File for information about the HLA types). With this criteria we obtained 885 TCRs specific to seven epitopes (pp65_495-503_, BMLF1_280-288_, BRLF1_109-117_, M1_58-66_, NS3_1073-1081_, NS3_1406-1415_, NS4B_214-222_). Fig 4B and S9 Fig show that using all CDR*β*s instead of only CDR3*β* is beneficial also in this case and that the predictive performance remains good. In fact, the mean AUROC scores in this case (0.794 and 0.821 when only CDR3*β* and all CDR*β*s were used) are higher than the mean AUROC scores obtained for the same seven HLA-A*02 restricted epitopes, when TCRs for all 22 epitopes were utilized (0.746 and 0.786, respectively), see S9 Fig. This analysis supports our finding that CDRs other than CDR3 contain information that is useful for predicting TCR-epitope recognition. A summary of all prediction results is shown in S1 File.

### Evaluation on TCR data from independent studies

The above experiments have demonstrated TCRGP’s accuracy when the classifier has been tested with TCR sequences from new subjects. Next we tested TCRGP’s robustness to generalize between independent studies. Specifically, we tested how TCRGP performs when trained on the epitope-specific TCR sequences from one study and tested on sequences from another independent study. We performed this experiment for the nine epitopes from the VDJdb data that have a study with at least 50 TCRs for training the model and another independent study with at least 10 TCRs for testing. When there were multiple studies that fulfilled this criteria, we chose the two studies with most unique TCRs for training and testing, respectively. When only CDR3*β* was utilized, we obtained a mean AUROC score of 0.813, and with all CDR*β*s the AUROC score was 0.865, see S1 File for AUROC scores for all these epitopes and information of the number of TCRs for each study.

We also performed a leave-one-study-out cross-validation for all 10 epitopes that have always at least 50 TCRs for training and 10 TCRs for testing when divided to folds based on the study they were obtained from. For this experiment we only used studies with at least five TCRs. We achieved AUROC scores of 0.838 and 0.861 when utilizing CDR3*β* and all CDR*β*s, respectively (see S1 File).

Additionally, we trained a classifier for COVID-19 epitope S-protein_269-277_ (YLQPRTFLL) with the 352 epitope-specific TCR*β*s from the recent study of Shomuradova et *al.* [34] and tested the classifier with the 415 in-frame S-protein_269-277_-specific TCRs from the ImmuneRACE study launched by Adaptive Biotechnologies and Microsoft (https://immunerace.adaptivebiotech.com, June 10, 2020 dataset). The immuneRACE data did not have sufficient information of the V*β*-gene for us to utilize all CDR*β*s, so the classifier was trained and tested only with CDR3*β*. Nonetheless, we achieved an AUROC score of 0.895 on the independent test data.

These experiments show that TCRGP is robust to overfitting and has high accuracy also when testing with TCRs from independent studies.

### Leveraging TCRGP in single-cell RNA+TCR*αβ*-sequencing data analysis

We next demonstrate how TCRGP can be utilized to implement a novel analysis of combined single-cell RNA and TCR*αβ* (scRNA+TCR*αβ*) sequencing data. Hepatocellular carcinoma (HCC) is one of the leading causes for cancer-related deaths worldwide [35]. Globally the predominant cause of HCC is considered to be Hepatitis B virus (HBV) as half of the HCC patients are estimated to be chronic HBV carriers [36]. During the course of natural infection, HBV integrates itself into the genome of the hepatocytes and thus a proportion of the HCC cells expresses HBV antigens [37]. Therefore, the malignant cells could be targeted by HBV-specific T-cell clonotypes and the high-dimensional characterization of these clonotypes could be crucial in understanding the viral control of HBV-infection and its association to HCC. To address this previously unanswered question we used TCRGP to analyze a dataset of single-cell RNA and TCR*αβ* of T cells from HBsAg-positive HCC-patients from blood, non-malignant liver tissue and tumour tissue published by Zheng et *al.* [38] (the Zheng data, see Materials and methods for details).

**Fig 5:**
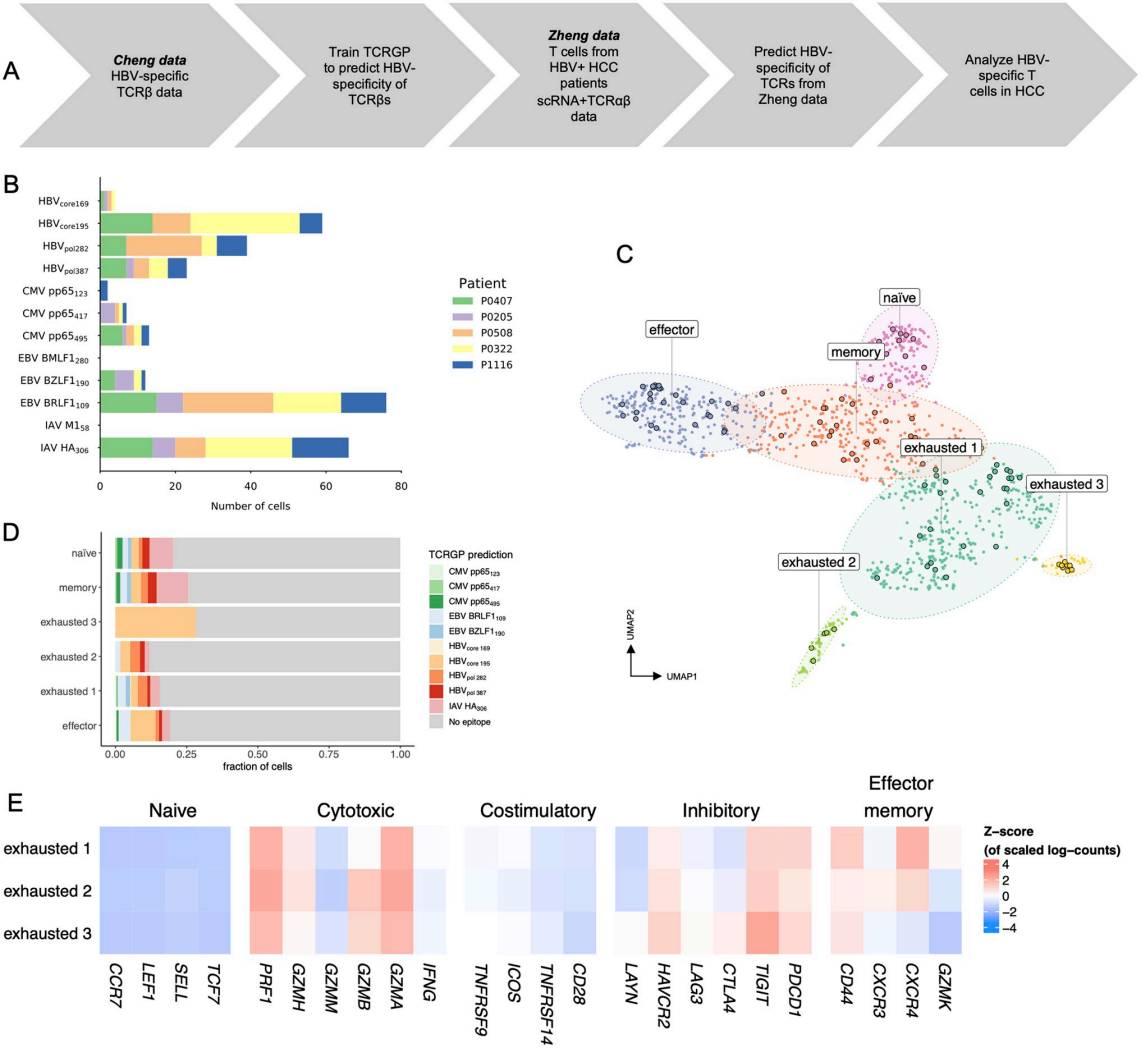
Analysis of HBV-specific T cells in HCC patients. **(A)** Schematics for the analysis of single-cell RNA and TCR*αβ* sequencing data using TCRGP and multimer-sorted data. **(B)** Numbers of cells predicted to recognize different epitopes by TCRGP with probability of at least 85%. HBV-reactivity was assessed by four different TCRGP classifiers trained against four different HBV-epitopes (HBV_core169_, HBV_core195_, HBV_pol282_, HBV_pol387_). Other predictions were made using the models trained with the VDJdb data. **(C)** Dimensionality reduced representation (UMAP) of the 1189 CD8+ T cells from HBV+ HCC-patients from peripheral blood, normal adjacent tissue and tumour tissue. Encircled dots represent the T cells predicted to be HBV-reactive by TCRGP. **(D)** The frequencies of T cells predicted to recognize different HBV-epitopes in each cluster. **(E)** Z-score normalized mean expressions of known canonical markers to assess CD8+ cell phenotypes (naïve, cytotoxic, costimulatory inhibitory, and effector memory markers) in the three different exhausted cell clusters. Exhausted 3 was predicted to be enriched for HBV-targeting T cells (p = 3e-06, p.adj = 0.001).

We utilized HBV-reactive T cell populations mapped by Cheng et *al.* [39] (the Cheng data) to train TCRGP classifiers (see Materials and methods for details) to enable prediction for the unselected TCR repertoire in the Zheng data against the four epitopes (HBV_core169_, HBV_core195_, HBV_pol282_, HBV_pol387_) (Fig 5A).

Of the 789 CD8+ cells from Zheng data analyzed with TCRGP, 108 cells (13.688%), were predicted to be reactive against any of the four HBV epitopes with at least a TCRGP-based probability of 85%, most of which against HBV_core195_-epitope (59 cells) (Fig 5A–5C). On the contrary, 176 cells were predicted to be reactive against common viruses (CMV = 22 (three epitopes), EBV = 88 (three epitopes) and Influenza A = 66 cells (two epitopes)) (Fig 5B), suggesting that HBV was the most common target for antigen-specific T cells in HCC patients.

After unsupervised clustering of the CD8+ cells’ scRNA-seq data, we received 6 different phenotypes that were similar to the phenotypes described by Zheng et *al.* [38], but had the exhausted cells divided into 3 different clusters instead of one (naïve, effector, memory, exhausted 1, exhausted 2 and exhausted 3) (Fig 5C). Interestingly, cells in exhausted 3 cluster showed the highest enrichment of the clonotypes targeting HBV_core195_-epitope (Fisher’s exact test p = 3e-06, Benjamini-Hochberg corrected for multiple testing p.adj = 0.001), but not to any other epitope-specific clonotypes (Fig 5D and 5E). Enrichment of HBV targeting clonotypes was not significant with more stringent TCRGP prediction probabilities possibly due to small number of cells. By calculating exhaustion score for each T cell, we found that exhausted 3 cluster was the most exhausted (Mann-Whitney U test against exhausted 2 p = 0.003, against exhausted 1 p = 0.002) and the least cytotoxic cluster (p = 0.02 and p = 1e-05). Further, gene-level analysis showed high expression of *TIGIT* and *HAVCR2* (encoding TIM-3), which have been associated with late-stage exhaustion after long antigen exposure. Upregulated pathways for exhaustion cluster 3 were IL2-STAT5 signaling pathway (exh3 vs exh1 q = 0.022 and exh3 vs exh2 q = 0.000) and myogenesis pathway (q = 0.016 and q = 0.001).

In summary, TCRGP was able to identify a T cell cluster that was enriched with HBV-targeting clonotypes, which was the most exhausted and least functional. This could provide a mechanism for loss of immunological control against HBV after chronic antigen stimulation which could aid in pathogenesis towards HCC. The implementation for the analysis and the datasets used are available at https://github.com/janihuuh/tcrgp_manu_hcc.

## Discussion

In this paper we have demonstrated that when we have sufficient amount of experimentally produced epitope-specific TCR-sequencing data available for training a classifier, TCRGP can predict with a relatively high accuracy if previously unseen TCRs recognize an epitope. However, the exact number of TCRs required to achieve a certain level of accuracy varies greatly between the different epitopes. This likely reflects the fact that different epitopes can be more selective in choosing their TCR interactions. In other words, TCRs that recognize one epitope can be more diverse than the TCRs that recognize another epitope [10], and if the TCRs are very heterogeneous, it requires more sampling to get a representative sample of these TCRs for the model training. In addition, we have shown that also CDRs other than CDR3*β* can provide useful information for the classification task, although it depends on the epitope in question which of the CDRs are most important. Although computational methods cannot replace experimental measurements in determining exact epitope-specificities of TCRs, they may be used to complement them for example when analyzing existing unselected TCR repertoire data or to guide experimental designs for *ex vivo* measurements.

In this work we have provided a comprehensive analysis of current epitope-specificity prediction algorithms on a large set of publicly available epitope-specific TCR sequences. With the currently available epitope-specific TCR sequence data we have been able to come this far, but as more data becomes available with modern high-throughput techniques presented recently [40, 41], new possibilities will rise. With a larger variety of pMHC complexes and TCRs that recognize them, we hope to be able to better consider the cross-reactivity of TCRs, similarities between different epitopes, and the significance of the HLA-types of the MHC proteins presenting the epitopes and perhaps even predict if a TCR can recognize a previously unseen epitope. As the proposed GP formalism has been shown to scale to very large data sets with up to billion data points [42], it should be well suited for the arising challenge.

The previous supervised algorithms developed are presented in the case of epitope-specific data, but we believe that to answer clinically relevant questions we need to address the unselected repertoire data which is far more numerous in size and more easily produced. Therefore we presented a novel workflow for analysis of scRNA+TCR*αβ* data in a clinically relevant question, showing the power of determining the epitope-specifity *in silico* to reveal underlying transcriptomic heterogeneity of the epitope-specific T cells, which has been previously difficult to perform. As the number of scRNA+TCR*αβ* and conventional TCR*β* sequencing data in clinical settings is increasing [43–50], we expect that models like ours can be applied to a variety of research questions where exhaustive *ex vivo* pMHC-multimer assays are not feasible. In conclusion, we propose that TCRGP could be useful in the diagnosis and follow-up of infectious diseases, in autoimmune disorders and cancer immunotherapy.

## Materials and methods

### Data

#### T cell receptor sequences

Our experiments focus on TCRs formed by a pair of *α*- and *β*-chains, as those are the most common type of TCRs [51]. The CDR3 sequence is formed by V(D)J recombination, but CDR1, CDR2, and CDR2.5 sequences are determined completely by the V-gene and allele [3]. We align the CDR3 sequence by adding a gap at the top of the loop, following the IMGT numbering [11]. Dash et *al.* [10] provide a table of all V-gene and allele combinations and the corresponding CDR1, CDR2, and CDR2.5 amino acid sequences aligned according to IMGT definitions [11]. Our method can utilize the aligned amino acid sequences of all these CDRs from either one or both of the *α*- and *β*-chains of the TCR. Fig 1 shows a few examples of TCR sequences and their alignment.

#### Datasets

In our experiments, we use a data set collected by Dash et *al.* [10] (from hereon referred to as Dash data), which contains epitope-specific paired TCR*α* and TCR*β* chains for three epitopes from humans and for seven epitopes from mice, see Table 3 for details.

We also gather a new data set (VDJdb data) from VDJdb (http://https://vdjdb.cdr3.net, downloaded 9th October 2018) [16], which is a database that contains TCR sequences with known antigen specificity. Every entry in VDJdb has been given a confidence score between 0 and 3 (0: critical information missing, 1: medium confidence, 2: high confidence, 3: very high confidence). We constructed our data set so that we selected all epitopes that have at least 50 TCR*β* sequences with a confidence score at least 1 and found 22 such epitopes. Twenty of these epitopes are MCH class I restricted and two are MHC class II restricted and have varying HLA-types, see Table 3 for details. VDJdb also contains TCR*α* sequences, but since these are not in general paired with the corresponding TCR*β* sequences, we chose to only experiment with the TCR*β* sequences. The VDJdb data and the Dash data have some overlap for TCRs specific to three epitopes: In the VDJdb data 34 (27 unique) of the 413 (242 unique) TCRs for pp65_495-503_, 30 (27) of 299 (152) for BMLF1_280-288_, and 74 (61) of 239 (138) for M1_58-66_ can also be found from the Dash data.

For the training and testing of the models, we also required some background TCRs that we do not expect to recognize the epitopes in our data sets. For this purpose, we randomly sampled the required amount of TCRs from sets of background TCRs constructed by Dash et *al.* [10]. They report that the human *α*- and *β*-chains have been obtained from Howie et *al.* [52], who have collected blood from two healthy adults. The mouse *α*-chains they have gathered from short read archive (SRA) projects SRP010815 [53], and SRP059581 [54] and mouse *β*-chains from SRA projects SRP059581 [54], SRP015131 [55], and SRP004475. To create paired *α*- and *β*-chains they randomly paired the unpaired *α*- and *β*-chains from the repertoires for the corresponding organism.

#### Construction of training and test sets

For the evaluation of the methods developed by us and others, we needed to divide our data sets for training and testing. Both of the data sets we use determine the subjects from whom the TCRs in the data have been obtained from. We therefore chose to use leave-one-subject-out (LOSO) cross-validation, where we leave out all TCRs from one subject, train the model with all the other TCRs, test it with the TCRs left out, and repeat this for all subjects. The epitope-specific TCRs are always complemented with an equal number of background TCRs in both training and test sets, except for the experiments presented in Section Discriminating between epitope-specific TCRs, were we use only the VDJdb data. We thought this would be the most realistic procedure for the evaluation, as this is likely how these kinds of models will be applied to new data: A model is trained with some set of TCRs and then predictions would be made for TCRs sequenced from an individual from who we have not seen any TCR sequences beforehand. We have chosen to create separate models for each epitope, as this approach automatically handles potential cross-reactivity between different epitopes—we only have to select such TCRs for training the model that we can assume to recognize that one epitope or to not recognize that epitope. If we then want to predict TCR’s specificity to one or several epitopes, we can apply one or several models and get separate predictions for each of the epitopes.

In the Dash data the average number of TCRs per fold varied between 6 (for pp65_123-131_) and 22 (for M45_985-993_), and in the VDJdb data between 3 (for p24_30-40_) and 45 (for NS4B_214-222_). The number of subjects, TCRs and unique TCRs for each epitope can be found from Table 3.

The VDJdb data contains TCR sequences from multiple studies, many of which have used same conventions for naming their subjects. Therefore we used the combination of the PMID of the publication and the subject id as the subject identifier. For two epitopes, p24_223-231_ and NS3_1406-1415_ there were very few separate subjects, only one and four, respectively. With these epitopes we used 5-fold cross-validation instead of the LOSO cross-validation.

We also evaluated the methods using leave-one-out (LOO) cross-validation with only unique TCR sequences. With LOO, the model is trained with all but one TCR and then tested with the left out TCR, and this procedure is repeated for each TCR. This way, we could utilize the maximal amount of training samples and also evaluate the model performance solely on new TCRs. We considered a TCR to be unique when it consists of a unique combination of CDR3 amino acid sequences and V-genes from both *α*- and *β*-chains, when both are available, and from the *β*-chains, when only TCR*β* is utilized.

#### TCR repertoire diversity

To estimate the diversity of the epitope-specific TCRs for each epitope, we developed a diversity measure following the example of Dash et *al.* [10]. The Simpson’s diversity index was then generalized to account for the similarity of TCRs by utilizing the Gaussian kernel function as follows:

$$\begin{array}{c} \text{diversity}={\left(\frac{\sum_{i=0}^{N-1}\sum_{j=i+1}^{N}\sigma^{2}\exp(-\frac{\left|\right|x_{i}-x_{j}{\left|\right|}^{2}}{2l^{2}})}{\frac{1}{2}\left(N-1\right)N}\right)}^{-1}. \end{array}$$ (1)

Here *σ*^2^ is the kernel variance and *l* is the lengthscale of the Gaussian kernel used by TCRGP, and **x**_*i*_ and **x**_*j*_ are feature vectors for the TCRs *i*, *j* ∈ [1, *N*]. The kernel variance and lengthscale were set to the average values used for the 22 epitopes in the VDJdb data (*σ*^2^ = 5.52, *l* = 2.50).

### TCRGP classifier

#### Sequence representation

Computational methods require the data to have some presentation, that they can utilize. Character sequences with variable lengths often provide some challenges as many methods rely on numerical inputs of fixed sizes. One solution is to compare subsequences of same length instead of the complete sequences, which is what for example Generic String kernel (GSkernel) does [56]. However, by aligning the sequences more approaches become applicable. According to the IMGT definitions [11] CDR3s can be aligned by introducing a gap in the middle of the sequence (i.e. top of the loop). Alignments for CDR1s, CDR2s, and CDR2.5s can be found from http://www.imgt.org. When the sequences are aligned, all the sequences within a CDR class (1, 2, 2.5 or 3) have the same length (see Fig 1A).

We observe sequences *a*_1_ *a*_2_⋯*a*_*L*_ of amino acids $a_{j}\in A=\{A,R,N,\ldots ,-\}$ at aligned positions *j* = 1, …, *L*. The alignment guarantees that all sequences have the same possibly padded length *L*. We encode the amino acids *a* with global feature vectors $\phi (a)\in {\mathbb{R}}^{D}$ that associate a *D*-length real-valued code with each of the 21 amino acids including the gap symbol. The sequences are then encoded as data vectors **x** by concatenating the *L* feature vectors into a *D* ⋅ *L* length column vectors $x=(\phi (a_{1}{)}^{T},\ldots ,\phi (a_{L}{)}^{T}{)}^{T}\in X$. We collect a dataset of *N* sequences into a matrix $X=(x_{1},\ldots ,x_{N}{)}^{T}\in {\mathbb{R}}^{N\times DL}$ with rows as sequences and columns as individual amino acid features in aligned order. Each sequence is associated with a class label *y*_*i*_ ∈ {0, 1} that indicates whether the sequence was epitope-specific or not. We collect the class labels into an output vector **y** = (*y*_1_, …, *y*_*N*_)^*T*^ ∈ {0, 1}^*N*^.

We can observe amino acid sequences of the four complementarity determining regions (CDR) {1, 2, 2.5, 3} for both the *α*- and *β*-chains from a single TCR. Sequence data for each chain and CDR combination has an individual alignment and sequence length. We denote the data as (**X**_*α*,1_, **X**_*α*,2_, **X**_*α*,2.5_, **X**_*α*,3_, **X**_*β*,1_, **X**_*β*,2_, **X**_*β*,2.5_, **X**_*β*,3_, **y**).

Substitution matrices such as BLOSUM62 [57] describe the similarity of each amino acid. We modified the BLOSUM62 to include also the gap used in alignments and scaled the matrix values between 0 and 1. The similarity of the gap and all amino acids were set to zero and the value on the diagonal was set to the smallest value on the diagonal. The resulting matrix $B\in {\mathbb{R}}^{21\times 21}$ is then positive semidefinite. We apply eigendecomposition **B** = **VSV**^*T*^, where the column vectors of **V** encode orthogonal projections of the amino acids on the rows. We use the row vectors of **V**, indexed by the amino acids *a* from the modified BLOSUM62, as our descriptions ***ϕ***(*a*) = **V**_*a*,:_ with a feature representation ***ϕ***(*a*)^*T*^**S*ϕ***(*b*) = [**B**]_*ab*_ for any two amino acids a,b∈A. It is possible to use also different substitution models and feature vectors obtained from different sources, or to even use the so-called one-hot-encoding, but here we relied only on the eigenvectors of the (gap-extended) BLOSUM62.

#### Gaussian process classification

We use Gaussian process (GP) classification [58] to predict if a TCR recognizes a certain epitope or not. Gaussian processes model Gaussian distributions of non-parametric and non-linear functions. We apply a link function to squash the function values to a range [0, 1] suitable for classification. GPs have a clear advantage of characterizing the prediction uncertainty with class probabilities instead of point predictions. GPs naturally model sequences through kernel functions focusing on sequence similarity as the explaining factor for class predictions.

We use a GP function *f* to predict the latent epitope-specificity *score* f(x)∈ℝ of a sequence **x**. A zero-mean GP prior

$$\begin{array}{c} f\left(x\right)∼GP(0,k\left(x,x^{\prime }\right)), \end{array}$$

defines a distribution over functions *f*(**x**) whose mean and covariance are

$$\begin{array}{cc} E[f(x)] & =0\\ \text{cov}[f\left(x\right),f\left(x^{\prime }\right)] & =k(x,x^{\prime }), \end{array}$$

where *k*(⋅, ⋅) is the kernel function. We use the standard squared exponential kernel on the vectorized feature representation,

$$\begin{array}{c} k\left(x,x^{\prime }|\theta \right)=\sigma^{2}\exp(-\frac{{\left(x-x^{\prime }\right)}^{T}\left(x-x^{\prime }\right)}{2\ell^{2}}), \end{array}$$ (2)

where *ℓ* is the length-scale parameter, *σ*^2^ is the magnitude parameter and *θ* = (*ℓ*, *σ*^2^). For any collection of TCR sequences **X** = (**x**_1_, …, **x**_*N*_), the function values follow a multivariate normal distribution

$$\begin{array}{c} p\left(f\right)=N\left(f|0,K_{XX}\right), \end{array}$$ (3)

where $f=(f(x_{1}),\ldots ,f(x_{N}){)}^{T}\in {\mathbb{R}}^{N}$ collects all function predictions of the sequences, and $K_{XX}\in {\mathbb{R}}^{N\times N}$ is the sequence similarity matrix with [**K**_**XX**_]_*ij*_ = *k*(**x**_*i*_, **x**_*j*_). The key property of Gaussian processes is that they couple all predictions to be dependent. The Gaussian process predicts similar epitope values *f*(**x**), *f*(**x**′) for sequences **x**, **x**′ if they are similar according to the kernel *k*(**x**, **x**′).

The latent function *f*(**x**) represents an unbounded real-valued classification score, which we turn into a classification likelihood by the probit link function Φ:ℝ↦[0,1],

$$\begin{array}{c} \Phi \left(f\right)=\frac{1}{\sqrt{2\pi }}\int_{-\infty }^{f}\exp(-\frac{1}{2}\tau^{2})d\tau . \end{array}$$ (4)

The joint model then decomposes into a factorized Bernoulli likelihood and Gaussian prior,

$$\begin{array}{c} p(y,f)=p(y|f)p(f) \end{array}$$ (5)

$$\begin{array}{cc} & =[\prod_{i=1}^{N}\text{Ber}\left(y_{i}|\Phi \left(f_{i}\right)\right)]\cdot N\left(f|0,K_{XX}\right), \end{array}$$ (6)

where *f*_*i*_ is a shorthand for *f*(**x**_*i*_). The objective of Gaussian process modelling is to infer the posterior distribution *p*(**f**|**y**), which is intractable for many non-Gaussian likelihoods. Additionally inferring the kernel hyper-parameters *θ* entails computing the marginalized *evidence*

$$\begin{array}{c} p(y;\theta )=E_{p(f;\theta )}\left[p\left(y|f\right)\right], \end{array}$$ (7)

which is also intractable in general and has a limiting cubic complexity $O(N^{3})$[58]. We tackle the scalability with sparse Gaussian processes [59] and the intractability with stochastic variational inference [32].

#### Variational inference for low-rank GP approximation

We consider low-rank sparse Gaussian processes by augmenting the system with *M* inducing *landmark* pseudo-sequences $z_{j}\in X$ with associated (label) function values $u_{j}=f(z_{j})\in \mathbb{R}$. We collect all inducing points into structures **Z** = (**z**_1_, …, **z**_*M*_)^*T*^ and **u** = (*u*_1_, …, *u*_*M*_)^*T*^. By conditioning the GP with these values we obtain the augmented Gaussian process joint model

$$\begin{array}{c} p(y,f,u)=p(y|f)p(f|u)p(u) \end{array}$$ (8)

$$\begin{array}{c} p(f|u)=N(f|Au,K_{XX}-AK_{ZZ}A^{T}) \end{array}$$ (9)

$$\begin{array}{c} p(u)=N(u|0,K_{ZZ}) \end{array}$$ (10)

$$\begin{array}{c} A=K_{XZ}K_{ZZ}^{-1}, \end{array}$$ (11)

where $K_{XX}\in {\mathbb{R}}^{N\times N}$ is the kernel between observed sequences, **K**_**XZ**_ is between observed and induced sequences and **K**_**ZZ**_ is between induced sequences. The matrix **A** projects the *M* inducing points to the full observation space of *N* sequences.

Next, we define a variational approximation for the inducing points,

$$\begin{array}{c} q(u)=N(u|m,S) \end{array}$$ (12)

$$\begin{array}{c} q(f)=\int p(f|u)q(u)du \end{array}$$ (13)

$$\begin{array}{cc} & =N(f|Am,K_{XX}+A\left(S-K_{ZZ}\right)A^{T}), \end{array}$$ (14)

where $m\in {\mathbb{R}}^{M}$ and $S⪰0\in {\mathbb{R}}^{M\times M}$ are free variational parameters to be optimized. It can be shown that minimizing the Kullback-Leibler divergence KL[*q*(**u**)||*p*(**u**|**y**)] between the approximative posterior *q*(**u**) and the true low-rank posterior *p*(**u**|**y**) is equivalent to maximizing the evidence lower bound (ELBO) [60]

$$\begin{array}{c} p(y)\geq \sum_{i=1}^{n}E_{q(f_{i})}\left[\log p\left(y_{i}|f_{i}\right)\right]-\text{KL}[q\left(u\right)||p\left(u\right)]. \end{array}$$ (15)

The log expectation is tractable for Probit likelihoods [61], while the KL term similarly has a closed form for two Gaussian densities.

Due to the small data regime we choose the optimal assignment of selecting **Z** = **X** and **u** = **y**, which corresponds to the full Gaussian variational approximation of [62], while for larger datasets the inducing landmark points can also be optimised [32]. We then optimize the ELBO (Eq 15) with respect to the variational parameters **m** and **S** as well as the kernel hyperparameters *θ*, that is, the lengthscales *ℓ*_*cr*_ and weights *w*_*cr*_.

Finally, predictions f_*_ of new test sequences $X_{*}\subset X$ follow a variational predictive posterior

$$\begin{array}{c} p(f_{*}|y)=\int p\left(f_{*}|u\right)p\left(u|y\right)du \end{array}$$ (16)

$$\begin{array}{cc} & \approx \int p\left(f_{*}|u\right)q\left(u\right)du \end{array}$$ (17)

$$\begin{array}{cc} & =N(f_{*}|A_{*}m,K_{X_{*}X_{*}}+A_{*}\left(S-K_{ZZ}\right)A_{*}^{T}), \end{array}$$ (18)

where A_*_ indicates projection from the landmark points **Z** to the new sequences X_*_. The predictive distribution is a Gaussian distribution for the latent test values f_*_, from which the distributions of the test labels can be retrieved through the link function. We have implemented our model using GPflow, a Python package for building GP models using TensorFlow [63, 64]. We utilized GPflow’s variational GP model (VGP) and scalable variational GP model (SVGP)[31, 32].

#### Multiple kernel learning

When a TCR binds to a pMHC, its CDR3s and CDR1s from both *α*- and *β*-chains are in direct contact with the peptide most of the time. CDR2s and CDR2.5s can contact the peptide, but they usually only interact with the MHC, see Glanville et *al.* [9] for an overview of contacts between different CDRs and peptides. Dash et *al.* [10] took this into account by giving fixed weights for the distances between amino acids within different CDRs, giving more weight to the CDR3. As it varies which CDRs can be in contact with different peptides, we did not want to determine the importance of these different CDRs beforehand, but instead created separate kernels for each CDR and let our model decide which of them are important. We define the kernel as a convex combination of the base kernels for the four CDR regions *r* and the two chains *c*,

$$\begin{array}{c} k(x,x^{\prime })=\sum_{r\in \{1,2,2.5,3\}}\sum_{c\in \{\alpha ,\beta \}}w_{cr}k_{cr}\left(x,x^{\prime };\theta_{cr}\right), \end{array}$$ (19)

where the weights *w*_*cr*_ ≥ 0 are non-negative. While we can utilize all CDRs from both chains, it is also possible to use any subset of them.

### Single-cell RNA+TCR*αβ*-sequencing data analysis

#### TCRGP classifiers for HBV-epitopes

Recently, Cheng et *al.* [39] mapped HBV-reactive T cell populations by exhaustively screening the whole HBV genome with an HLA-class I restricted multiplexed pMHC-tetramer strategy and characterized T cells against four interesting HBV-epitopes from two antigens with TCR*β*-sequencing (the Cheng data). Utilizing the TCRs specific to HBV-epitopes HBV_core169_, HBV_core195_, HBV_pol282_, and HBV_pol387_ (here core refers to core protein and pol to polymerase protein) from the Cheng data and control sequences from Dash et *al.* [10] we trained a TCRGP classifier for each epitope. We utilized all epitope-specific TCRs from which we could determine also CDR1*β*, CDR2*β*, and CDR2.5*β* in addition to CDR3*β* and complemented these epitope-specific TCRs with the same amount of control TCRs. We considered TCRs which were predicted to recognize the epitopes with at least 85% probability as epitope-specific. The amounts of epitope-specific TCRs and AUROC scores obtained from leave-one-subject-out cross-validations for each epitope are shown in Table 4. We used TCRGP and VGP with all epitopes except for HBV_pol387_, with which we used SVGP with 700 inducing points due to the high number of samples.

**Table 4:** HBV-epitopes for which TCRGP classifiers were trained. The numbers of epitope-specific TCRs and subjects, and mean AUROC scores from leave-one-subject-out cross-validations are shown.

Epitope	Samples	Subjects	AUROC
HBV_core169_	699	9	0.756
HBV_core195_	588	12	0.847
HBV_pol282_	459	12	0.880
HBV_pol387_	1348	12	0.760

#### Single-cell RNA+TCR*αβ* data

Zheng et *al.* [38] published a dataset (the Zheng data) of single-cell RNA and TCR*αβ* of T cells from HBsAg-positive HCC-patients from blood, non-malignant liver tissue and tumour tissue. The unnormalized expression count data of T cells passing the quality control were fetched from GEO (GSE98638) along with the TCR*αβ*-sequences inferred from the full-transcript single-cell RNA-sequencing data and inferred phenotypic states as described by Zheng et *al.* As the TCR*β*-sequenced training data for HBV-specific epitopes was HLA-A restricted, we focused our analysis only on T cells capable of peptide recognition in HLA-A restricted manner, namely clusters CD8-LEF1, CD8-CX3CR1, CD8-LAYN and CD8-GZMK. The data was log-normalized to 10 000 counts per cell and scaled accordingly with the Seurat 3.0.2. [65] package for R 3.5.2.

#### Clustering

The highly variable genes (HVGs) were chosen to be the genes showing the highest mean to variance ratio (min expression = 0.5, max expression 3, min variance 0.5) with the FindVariableFeatures-function. The linear dimensionality reduction was calculated with PCA for the scaled expression matrix containing only HVGs. Non-linear dimensionality reduction was performed with UMAP for principal components that had standard deviation >2 using the standard parameters with the RunUMAP-function. To receive a better grouping for the selected cells, we used a graph-based clustering approach implemented in the Seurat tool. To find the shared nearest neighbor graph, the function FindNeighbors was used with the same amount of PCs as with UMAP. To determine optimal clustering, FindClusters-functions was used with several parameter values for the resolution parameter, ranging from [0.1, 3]. The optimal clustering was decided by agreement of grouping in the UMAP-embedding and the labels from clustering by visual interpretation.

The cytotoxic and exhaustion signatures for the clusters were calculated as cell-wise mean expression of cytotoxic (*NKG7*, *CCL4*, *CST7*, *PRF1*, *GZMA*, *GZMB*, *IFNG*, *CCL3*) and exhaustion genes (*CTLA4*, *PDCD1*, *HAVCR2*, *TIGIT*, *LAG3*). The difference between the signatures was assessed with Mann-Whitney U test for individual cells in clusters. Gene Set Enrichment Analysis (GSEA) between the clusters was performed on genes that were detected at least in 0.1% of the cells and had at least log fold-change of 0.01 between the tested cells. The gene list was ordered based on the fold-change. Overlap with HALLMARK-category was assessed and the False Discovery Rate (FDR) calculated while the number of permutations was 1000.

#### Enrichment analysis

The one-sided Fisher’s test for enrichment of epitope-specific T cells to different phenotypes was calculated independently for individual and pooled patients, epitopes and tissues. The obtained P-values were adjusted with Benjamini-Hochberg procedure for false-discovery.

## Supporting information

S1 Fig Mean AUROC scores for the Dash data using leave-one-subject-out cross-validation. TCRGP models (left column) and TCRdist models (right column) were trained using either only CDR3 or all CDRs from TCR*α*, TCR*β*, or both.
(PDF)

S2 Fig Distribution estimates and epitope-by-epitope method comparisons of mean AUROC scores for the Dash data using leave-one-out cross-validation with unique TCRs. **(A)** The blue parts of the violin plots illustrate the AUROC scores of predictions made by TCRGP for all the epitopes and the orange parts illustrate the AUROC scores obtained with TCRdist. Each point within a violin plot presents the mean AUROC score obtained for one epitope. The used chains (*α* and/or *β*) and CDRs (three or all) are indicated below each panel. **(B)** Comparison of AUROC scores obtained with TCRGP and TCRdist using only CDR3 from TCR*αβ*, TCR*β*, or TCR*α* for each epitope separately. The epitopes have been arranged in increasing order of AUROC scores obtained by TCRGP using CDR3 from *α*- and *β*-chains (blue line).**(C)** Comparison of AUROC scores obtained with TCRGP and TCRdist using all CDRs from TCR*αβ*, TCR*β*, or TCR*α* for each epitope separately. The epitopes have been arranged in increasing order of AUROC scores obtained by TCRGP using all CDRs from *α*- and *β*-chains (blue line).
(PDF)

S3 Fig Mean AUROC scores for the Dash data using leave-one-out cross-validation with unique TCRs. TCRGP models (left column) and TCRdist models (right column) we trained using either TCR*α*, TCR*β*, or both and either with only CDR3 or all CDRs.
(PDF)

S4 Fig Mean AUROC scores for the VDJdb data using leave-one-subject-out cross-validation. TCRGP models and TCRdist models (the first two columns) were trained using TCR*β* with either only CDR3 or all CDRs. RF models and DeepTCR models (the last two columns) were trained using the CDR3*β* and the V*β*-gene, from which the other CDRs can be derived from.
(PDF)

S5 Fig Distribution estimates and epitope-by-epitope method comparisons of mean AUROC scores for the VDJdb data using leave-one-out cross-validation with unique TCRs. **(A)** One violin plot presents the mean AUROC scores obtained with one method for all epitopes in the VDJdb data. Below each violin plot there is the name of the method used and in the brackets which CDRs have been used (3 for CDR3, all for CDR1, CDR2, CDR2.5, and CDR3). Each point within a violin plot presents the mean AUROC score obtained for one epitope. * RF (Random Forest TCR-classifier of De Neuter et *al.* [19]) could not produce predictions for all epitopes. The AUROC scores for RF have been obtained without these epitopes (8 and 4 epitopes were left out when only CDR3*β* was used and when also V*β*-gene was used, respectively, see S6 Fig). **(B)** Comparison of AUROC scores obtained with the different methods for each epitope separately. The epitopes have been arranged in increasing order of AUROC scores obtained by TCRGP using all CDR*β*s (orange line).
(PDF)

S6 Fig Mean AUROC scores for the VDJdb data using leave-one-out cross-validation. Only unique TCRs have been utilized. TCRGP models and TCRdist models (the first two columns) were trained using TCR*β* with either only CDR3 or all CDRs. RF models and DeepTCR models (the last two columns) were trained using the CDR3*β* and the V*β*-gene, from which the other CDRs can be derived from.
(PDF)

S7 Fig Learning curves. With each epitope from the VDJdb dataset, TCRGP models were trained using different numbers of unique epitope-specific TCRs, always complemented with the same number of control TCRs. For each point of the learning curve the model was trained with 100 random samples of the TCRs, using either CDR1, CDR2, CDR2.5, and CDR3 (blue curves), or only CDR3 (orange curves). The darker lines show the mean of the predictions and the shaded areas +/- the standard deviation for the 100 folds.
(PDF)

S8 Fig Diversity of TCRs within and between subjects. **(A)** Equation for computing the diversity between two subjects *s* and *t*. The diversity between multiple subjects can be computed similarly. **(B)** Scatter plot of diversities. Vertical axis shows diversity of epitope-specific TCRs between subjects and horizontal axis shows average diversity of TCRs within each subject. Diversities are computed for 21 epitopes from the VDJdb data (this data contained TCRs from only one subject for HIV-1 epitope p_223-231_, which was therefore left out from this figure). Diversities between subjects seem to be slightly larger than within subjects (on average 4.3% larger, Pearson correlation 0.82). **(C)** Pairwise diversities between subjects, where diversities within subjects are shown on the diagonal. Subjects are sorted by increasing diversity and only subjects with at least two TCRs have been included in this figure.
(PDF)

S9 Fig Comparisons with different control data. Comparisons with TCRGP with different control data using either all CDR*β*s (left column) or only CDR3*β*s (right column). 1: mean AUROC scores from leave-one-out cross validation when equal number of epitope-specific and control TCRs are used in training and testing (same as in S6 Fig). 2: Mean AUROC scores from stratified 200-fold cross validation when TCRs specific to other epitopes in the VDJdb data have been used as control data. 3: Otherwise same as 2, but only TCRs specific to epitopes restricted by MHC of type HLA-A*02 have been used for training and testing.
(PDF)

S1 File Result tables summarizing the prediction accuracy results presented in Figs 2B, 3B and 4A, and S1–S6 and S9 Figs, and a list of studies from which the VDJdb data consists of. (XLSX)

S2 File Benjamini-Hochberg corrected P-values from Wilocoxon signed rank tests for method comparisons with the Dash data and the VDJdb data. (XLSX)

S3 File Detailed information of the MHCs for different epitopes in the VDJdb data. (XLSX)
