# Supplementary figures and images for: Predicting recognition between T cell receptors and epitopes with TCRGP
Source: PLoS Comput Biol. 2021 Mar 25;17(3):e1008814. doi: 10.1371/journal.pcbi.1008814 (PMC8023491; doi:10.1371/journal.pcbi.1008814)

TCRGP

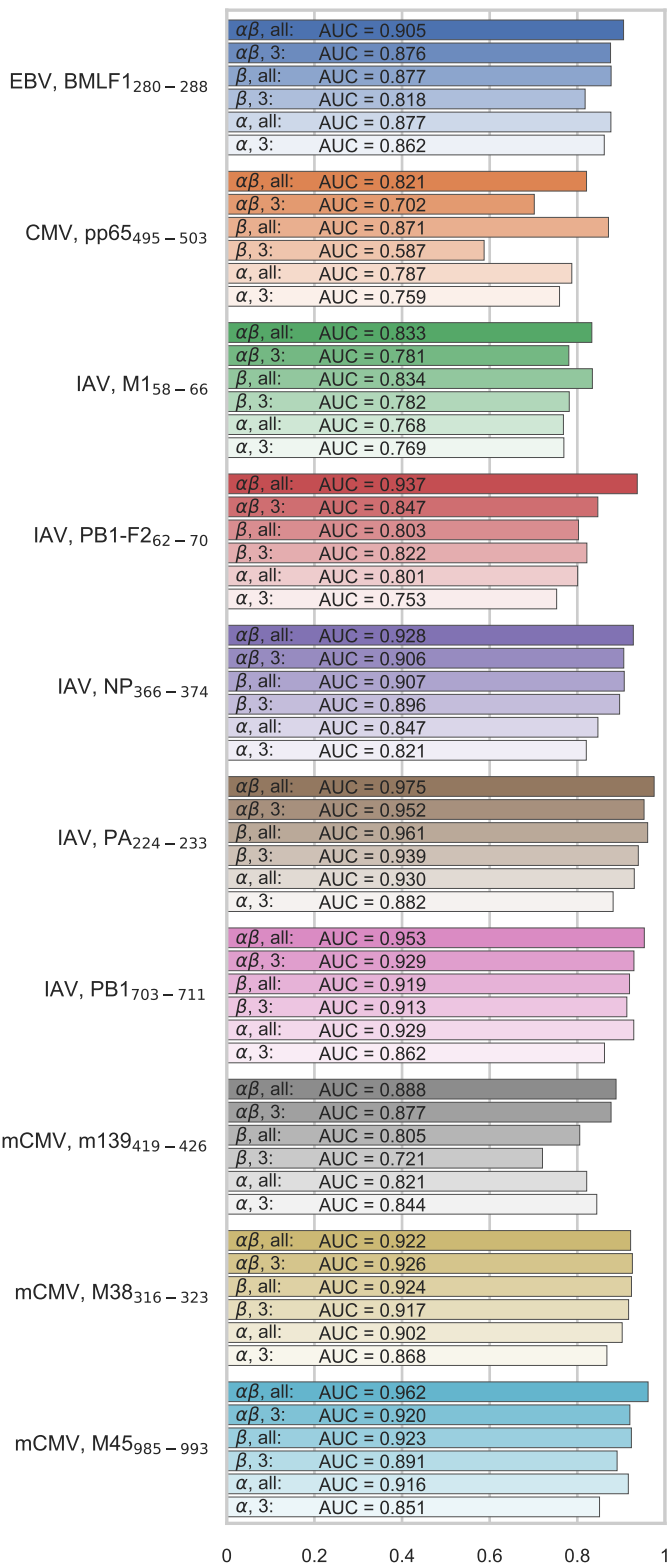

TCRdist

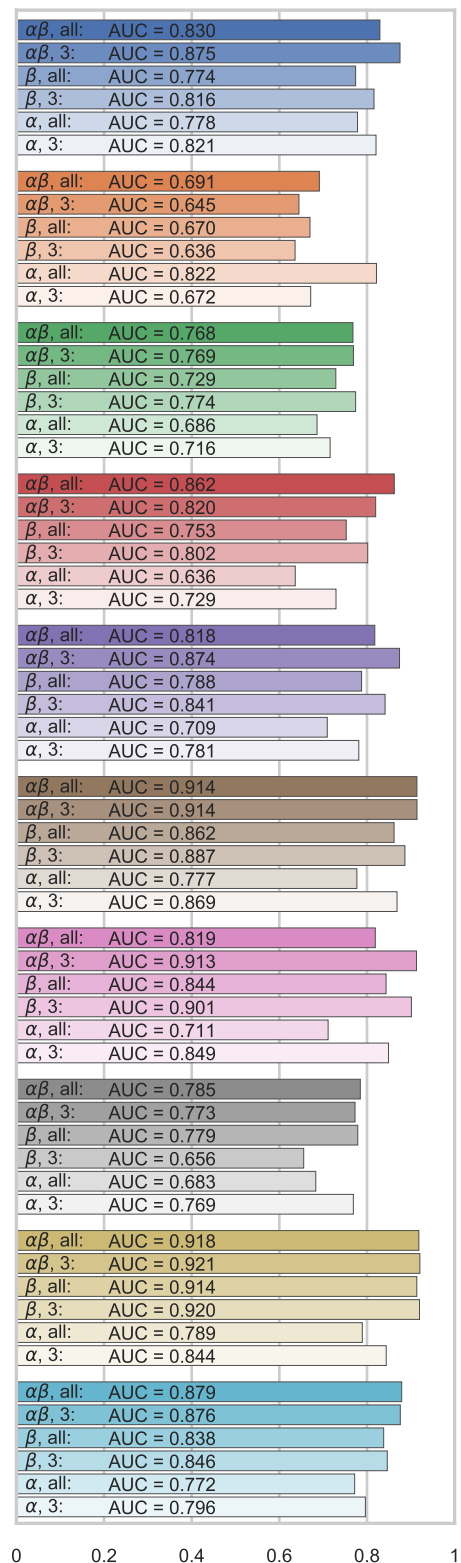

Supplement: S1 Fig — TCRGP models (left column) and TCRdist models (right column) were trained using either only CDR3 or all CDRs from TCRα, TCRβ, or both. (PDF) [file pcbi.1008814.s001.pdf]

A

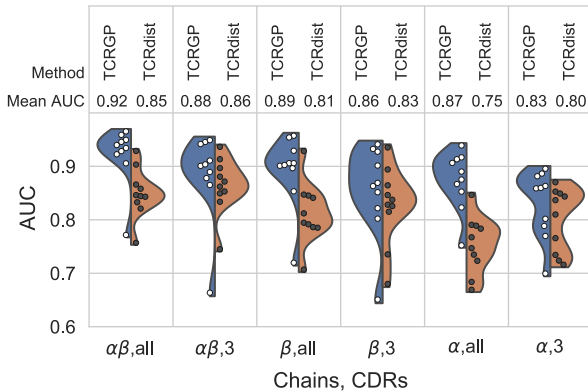

B

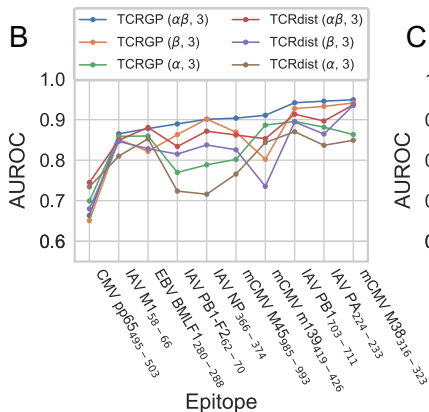

C

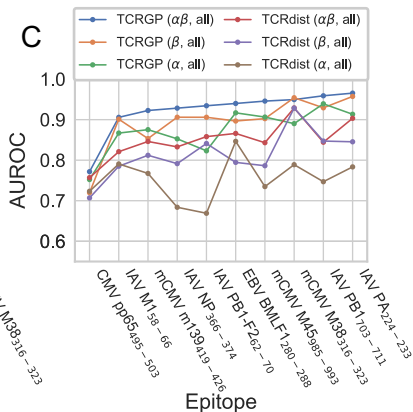

Supplement: S2 Fig — (A) The blue parts of the violin plots illustrate the AUROC scores of predictions made by TCRGP for all the epitopes and the orange parts illustrate the AUROC scores obtained with TCRdist. Each point within a violin plot presents the mean AUROC score obtained for one epitope. The used chains (α and/or β) and CDRs (three or all) are indicated below each panel. (B) Comparison of AUROC scores obtained with TCRGP and TCRdist using only CDR3 from TCRαβ, TCRβ, or TCRα for each epitope separately. The epitopes have been arranged in increasing order of AUROC scores obtained by TCRGP using CDR3 from α- and β-chains (blue line).(C) Comparison of AUROC scores obtained with TCRGP and TCRdist using all CDRs from TCRαβ, TCRβ, or TCRα for each epitope separately. The epitopes have been arranged in increasing order of AUROC scores obtained by TCRGP using all CDRs from α- and β-chains (blue line). (PDF) [file pcbi.1008814.s002.pdf]

TCRGP

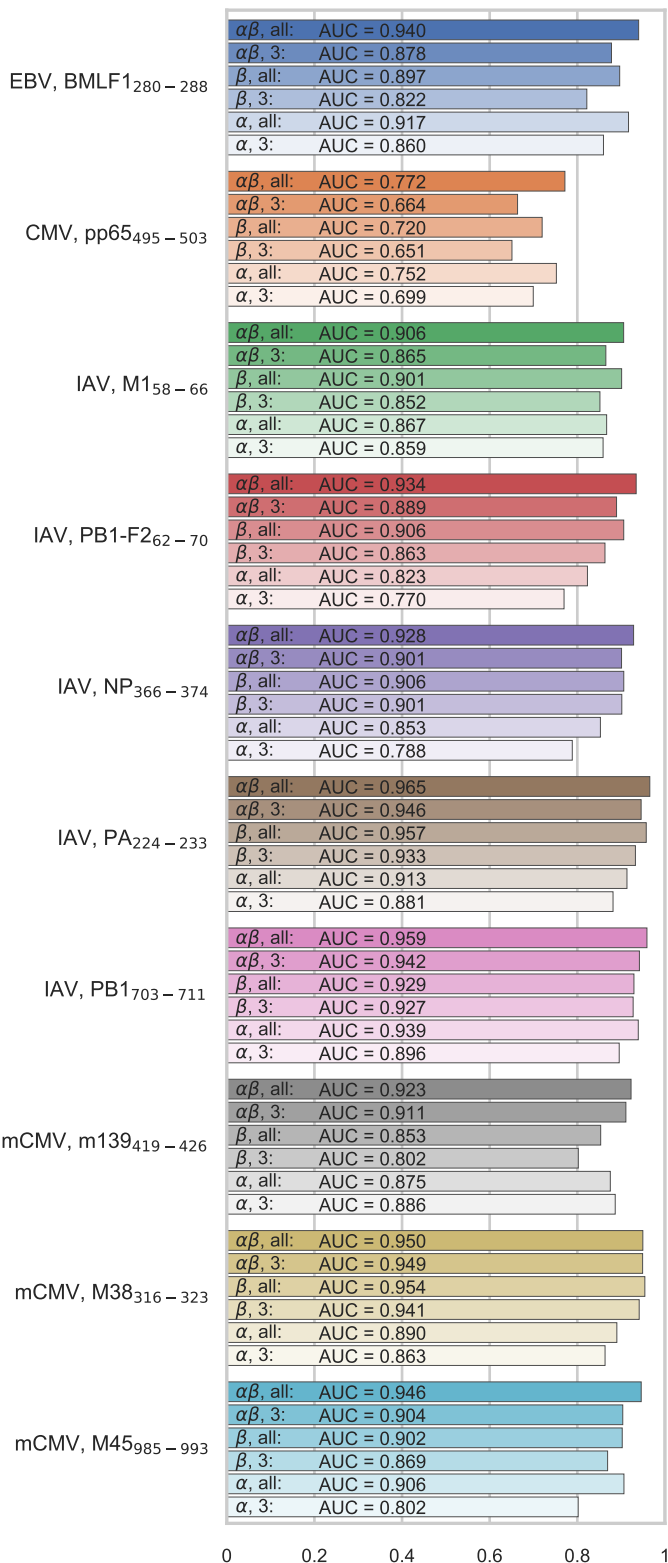

TCRdist

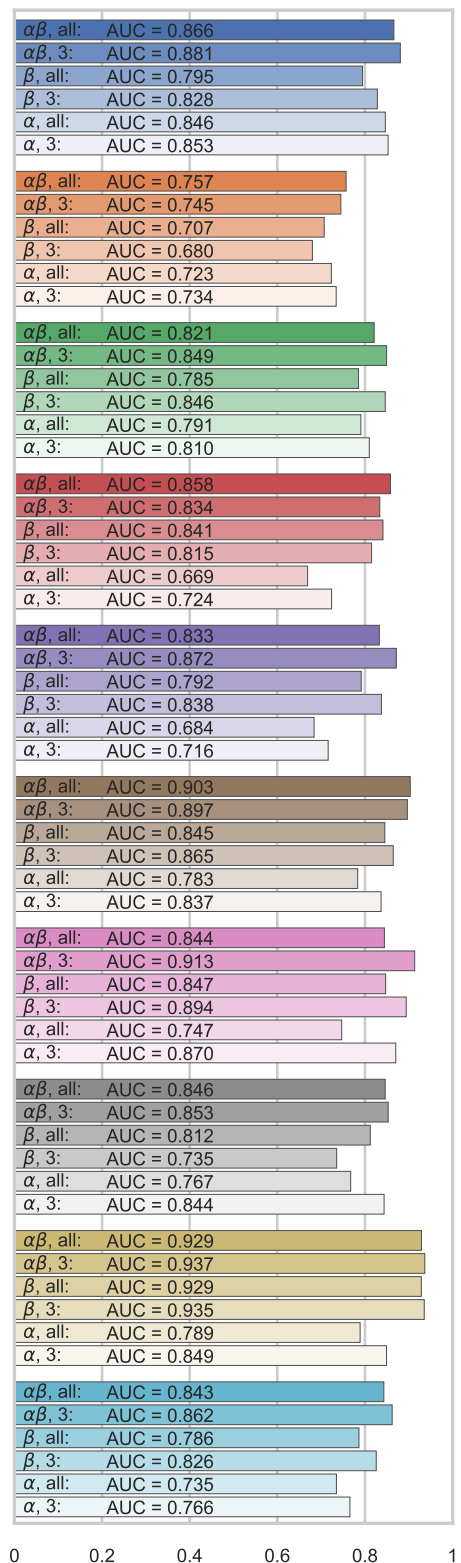

Supplement: S3 Fig — TCRGP models (left column) and TCRdist models (right column) we trained using either TCRα, TCRβ, or both and either with only CDR3 or all CDRs. (PDF) [file pcbi.1008814.s003.pdf]

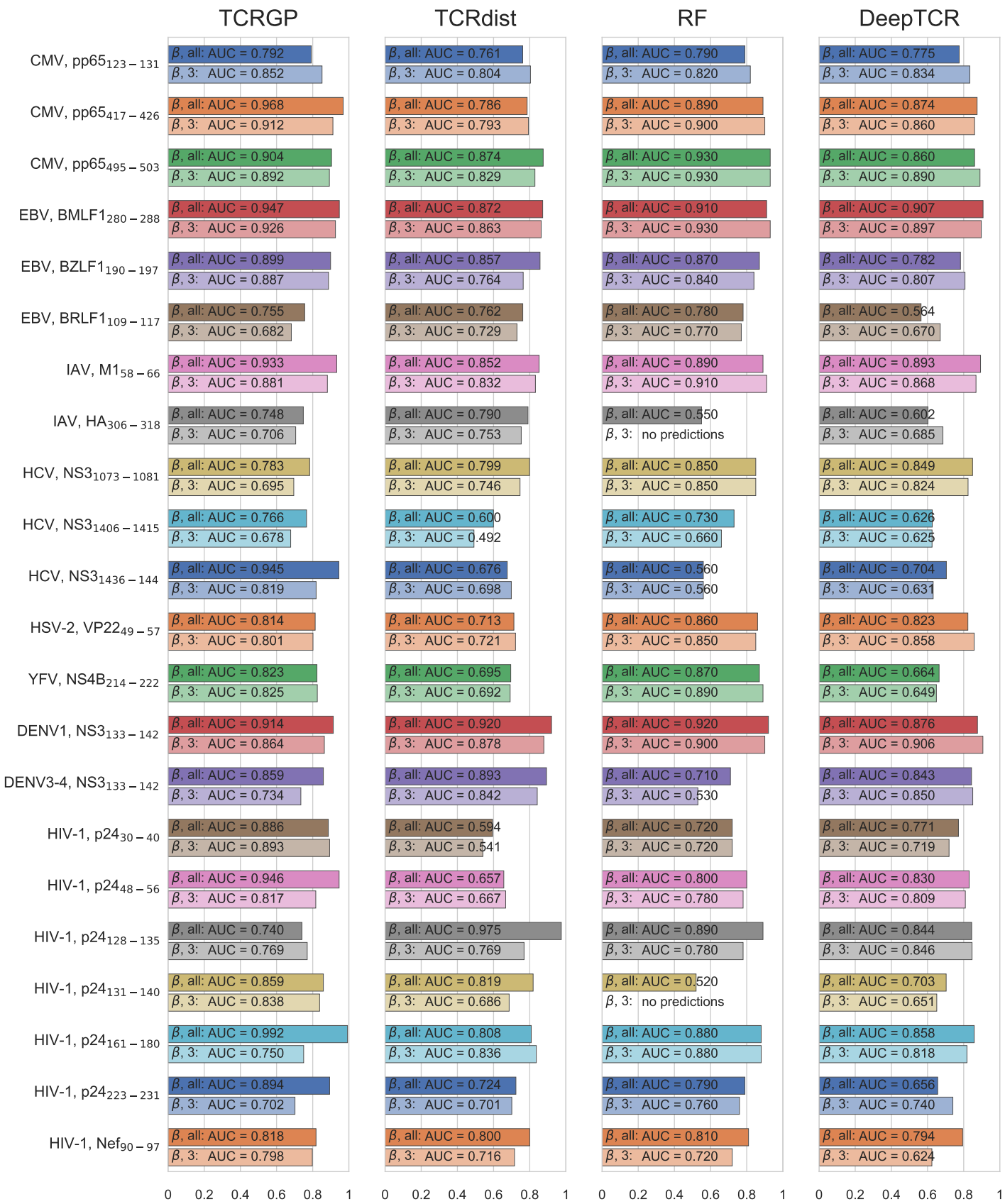

Supplement: S4 Fig — TCRGP models and TCRdist models (the first two columns) were trained using TCRβ with either only CDR3 or all CDRs. RF models and DeepTCR models (the last two columns) were trained using the CDR3β and the Vβ-gene, from which the other CDRs can be derived from. (PDF) [file pcbi.1008814.s004.pdf]

A

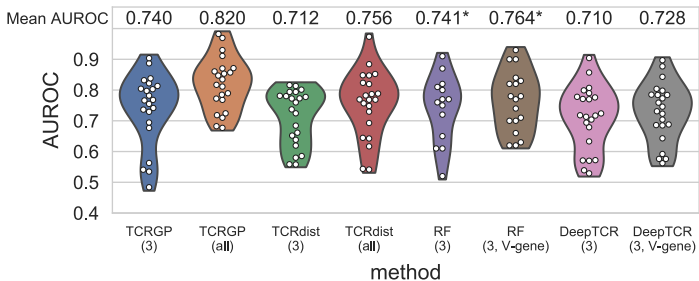

B

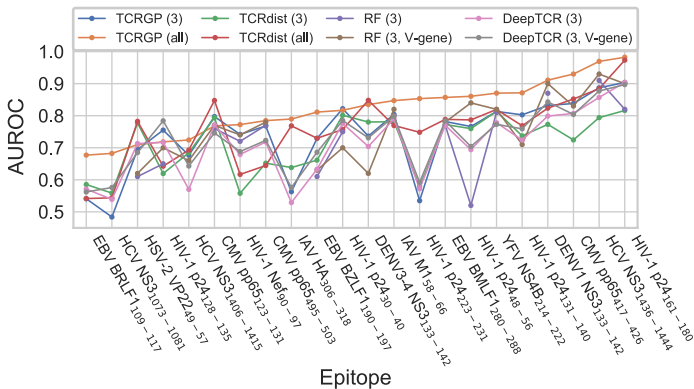

Supplement: S5 Fig — (A) One violin plot presents the mean AUROC scores obtained with one method for all epitopes in the VDJdb data. Below each violin plot there is the name of the method used and in the brackets which CDRs have been used (3 for CDR3, all for CDR1, CDR2, CDR2.5, and CDR3). Each point within a violin plot presents the mean AUROC score obtained for one epitope. * RF (Random Forest TCR-classifier of De Neuter et al. [19]) could not produce predictions for all epitopes. The AUROC scores for RF have been obtained without these epitopes (8 and 4 epitopes were left out when only CDR3β was used and when also Vβ-gene was used, respectively, see S6 Fig). (B) Comparison of AUROC scores obtained with the different methods for each epitope separately. The epitopes have been arranged in increasing order of AUROC scores obtained by TCRGP using all CDRβs (orange line). (PDF) [file pcbi.1008814.s005.pdf]

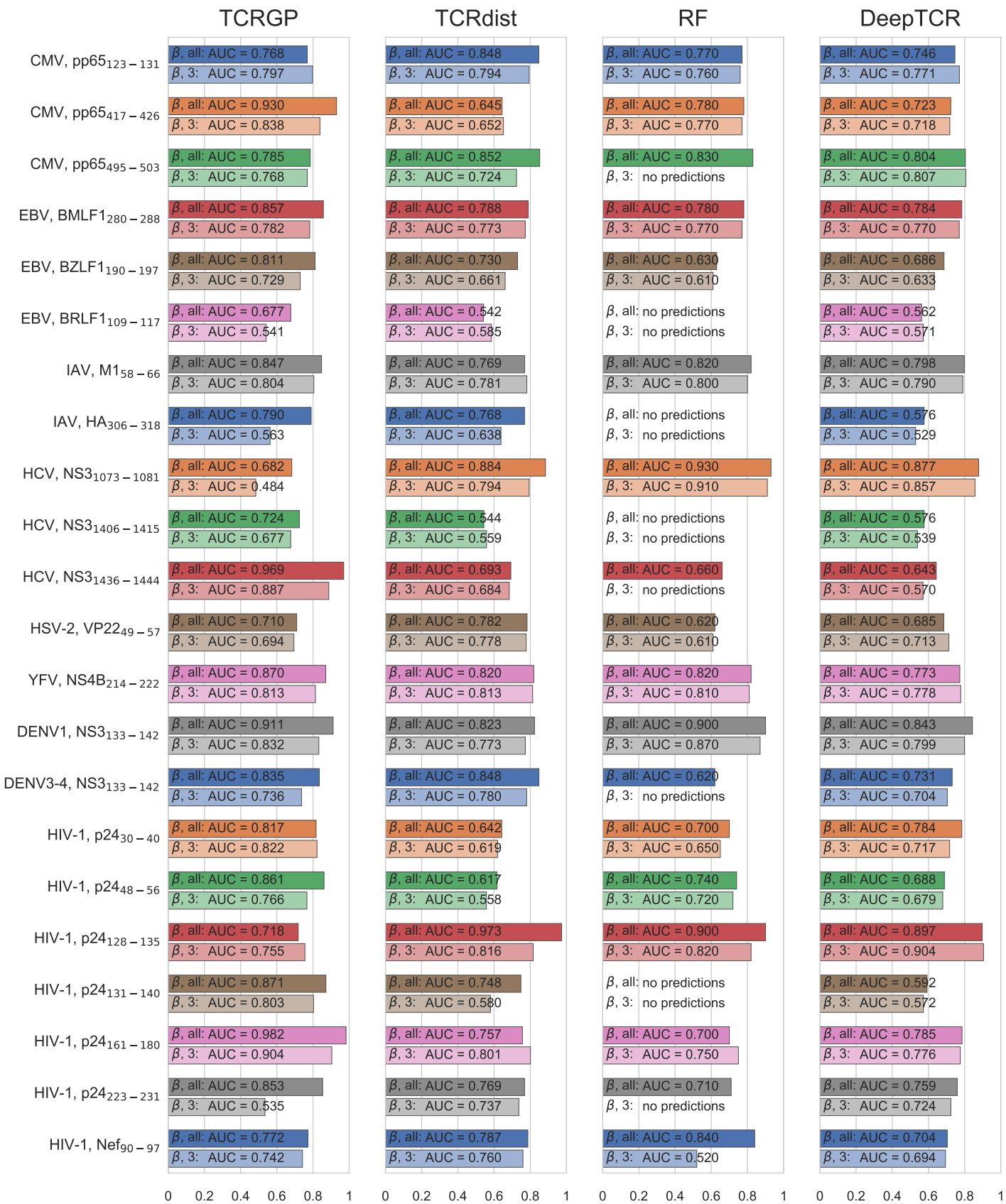

Supplement: S6 Fig — Only unique TCRs have been utilized. TCRGP models and TCRdist models (the first two columns) were trained using TCRβ with either only CDR3 or all CDRs. RF models and DeepTCR models (the last two columns) were trained using the CDR3β and the Vβ-gene, from which the other CDRs can be derived from. (PDF) [file pcbi.1008814.s006.pdf]

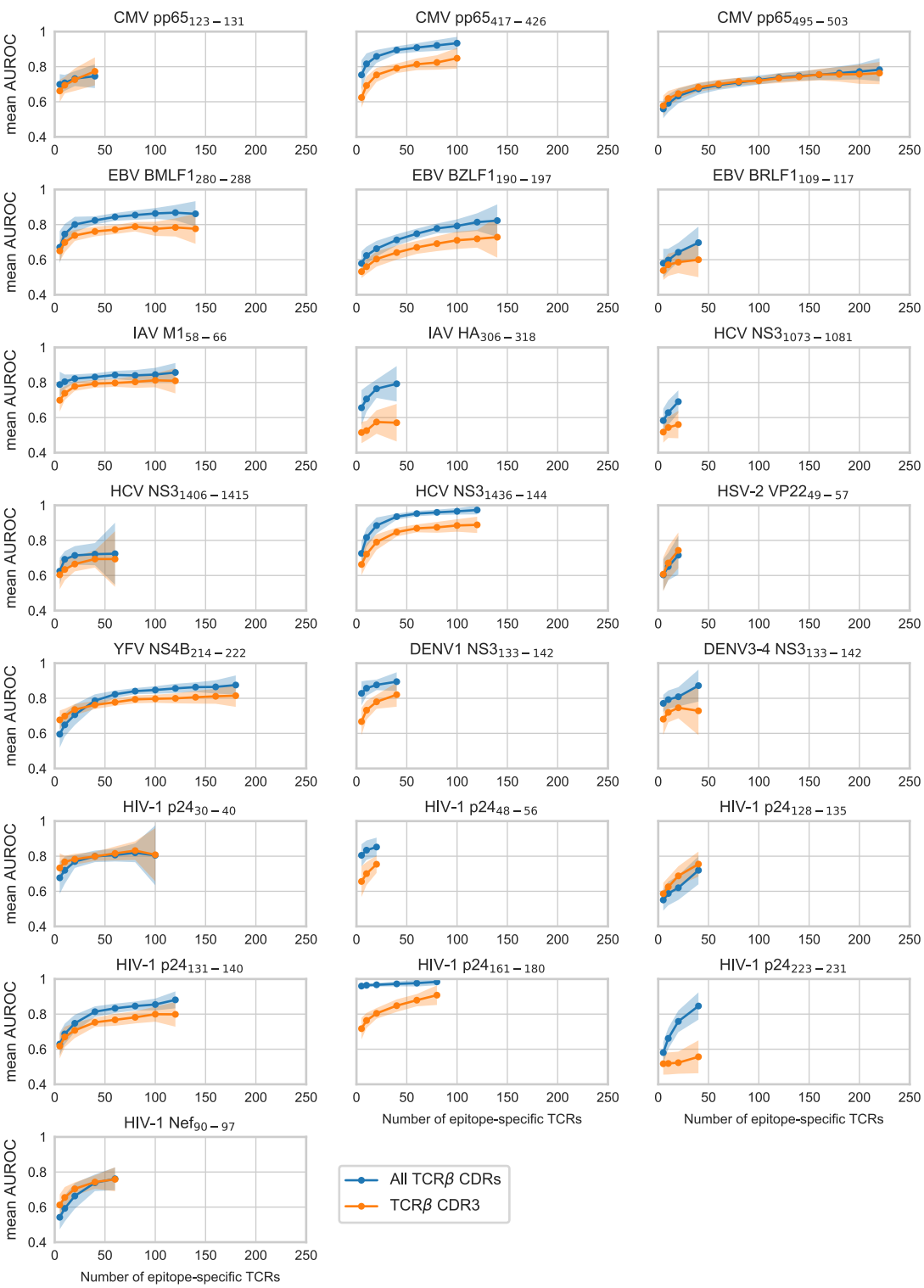

Supplement: S7 Fig — With each epitope from the VDJdb dataset, TCRGP models were trained using different numbers of unique epitope-specific TCRs, always complemented with the same number of control TCRs. For each point of the learning curve the model was trained with 100 random samples of the TCRs, using either CDR1, CDR2, CDR2.5, and CDR3 (blue curves), or only CDR3 (orange curves). The darker lines show the mean of the predictions and the shaded areas +/- the standard deviation for the 100 folds. (PDF) [file pcbi.1008814.s007.pdf]

all CDR $\beta$ s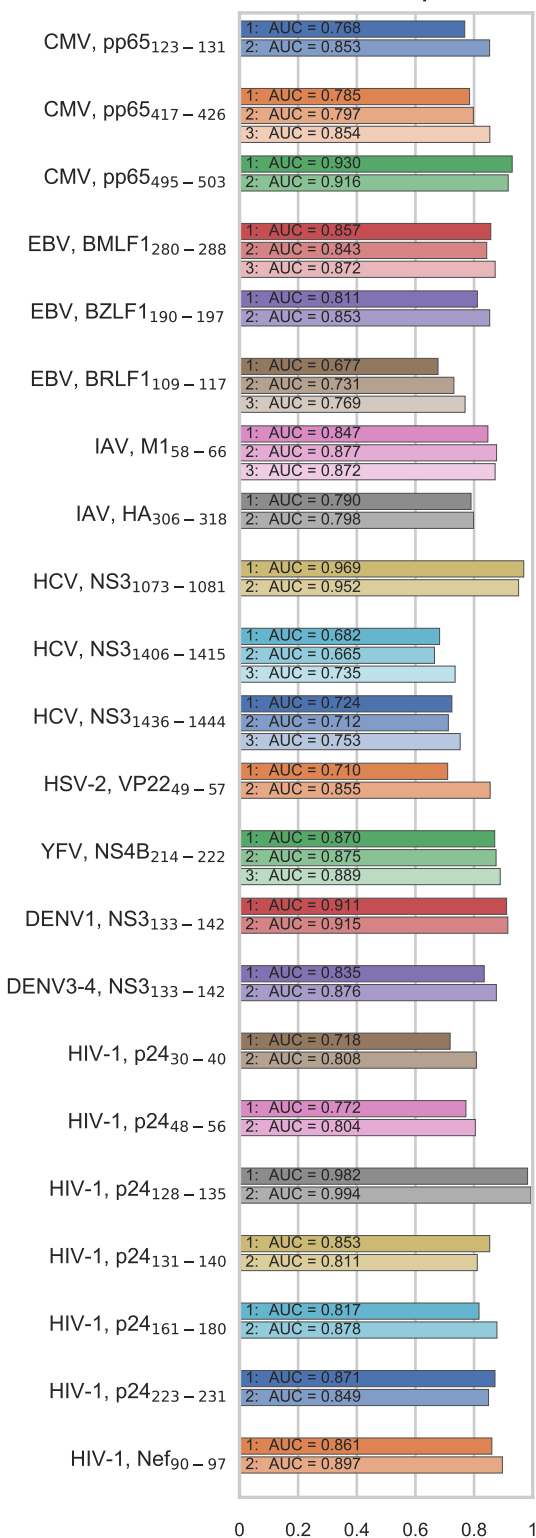CDR3 $\beta$ 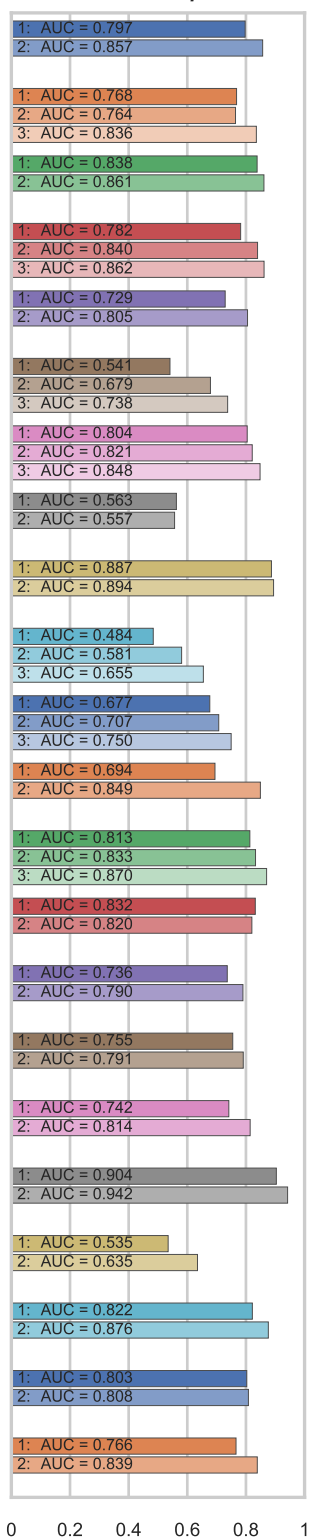

Supplement: S9 Fig — Comparisons with TCRGP with different control data using either all CDRβs (left column) or only CDR3βs (right column). 1: mean AUROC scores from leave-one-out cross validation when equal number of epitope-specific and control TCRs are used in training and testing (same as in S6 Fig). 2: Mean AUROC scores from stratified 200-fold cross validation when TCRs specific to other epitopes in the VDJdb data have been used as control data. 3: Otherwise same as 2, but only TCRs specific to epitopes restricted by MHC of type HLA-A*02 have been used for training and testing. (PDF) [file pcbi.1008814.s009.pdf]
