# Supplementary material for: Predicting recognition between T cell receptors and epitopes with TCRGP
Source: PLoS Comput Biol. 2021 Mar 25;17(3):e1008814. doi: 10.1371/journal.pcbi.1008814 (PMC8023491; doi:10.1371/journal.pcbi.1008814)

A

$$\mathrm{diversity}(s,t) = \left(\frac{\sum_{i=0}^{N_s} \sum_{j=0}^{N_t} \exp\left(-\frac{||\mathbf{x}_{s,i} - \mathbf{x}_{t,j}||^2}{2l^2} \right)}{N_s N_t} \right)^{-1}$$

$\mathrm{diversity}(s,t)$ describes the diversity between subjets s and t. N_s and N_t are numbers of TCRs $\mathbf{x}_{s,i}$ and $\mathbf{x}_{t,j}$ for the subjects s and t, respectively.

B

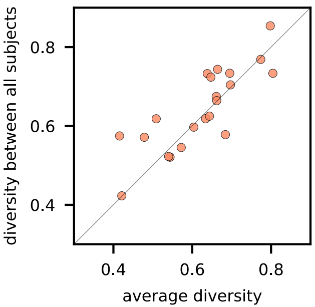

C

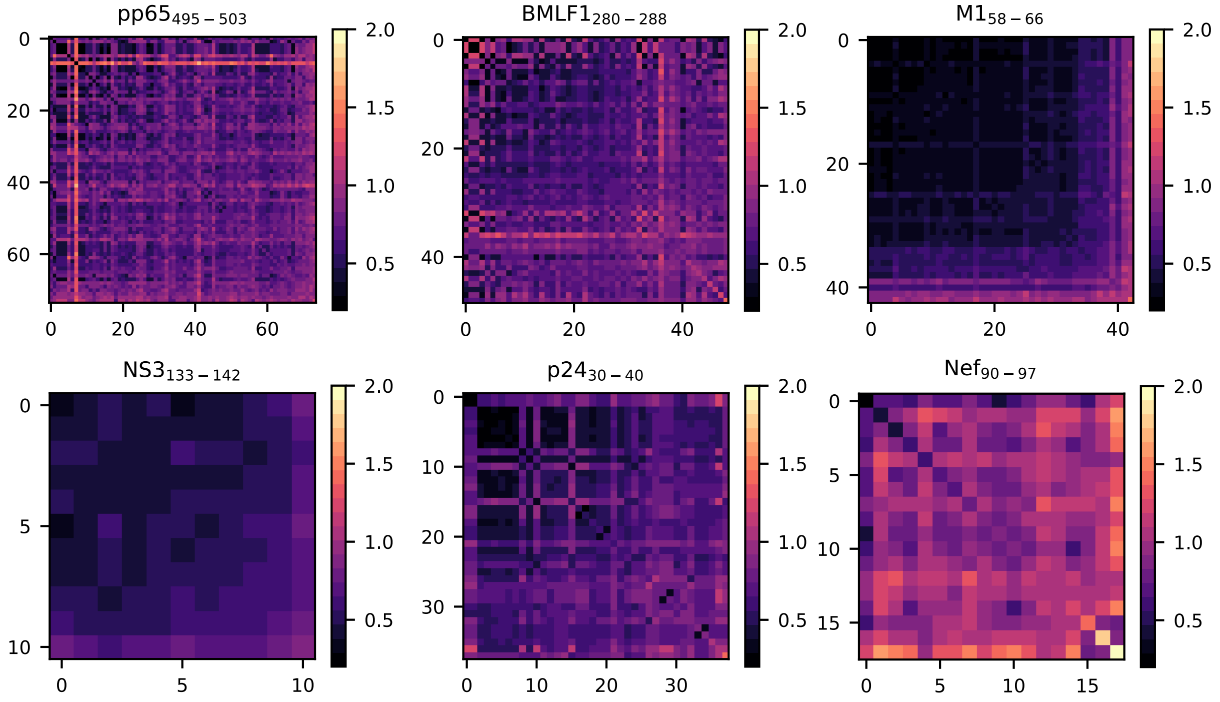

Supplement: S8 Fig — (A) Equation for computing the diversity between two subjects s and t. The diversity between multiple subjects can be computed similarly. (B) Scatter plot of diversities. Vertical axis shows diversity of epitope-specific TCRs between subjects and horizontal axis shows average diversity of TCRs within each subject. Diversities are computed for 21 epitopes from the VDJdb data (this data contained TCRs from only one subject for HIV-1 epitope p223-231, which was therefore left out from this figure). Diversities between subjects seem to be slightly larger than within subjects (on average 4.3% larger, Pearson correlation 0.82). (C) Pairwise diversities between subjects, where diversities within subjects are shown on the diagonal. Subjects are sorted by increasing diversity and only subjects with at least two TCRs have been included in this figure. (PDF) [file pcbi.1008814.s008.pdf]
